# Outside‐In Nanostructure Fabricated on LiCoO_2_ Surface for High‐Voltage Lithium‐Ion Batteries

**DOI:** 10.1002/advs.202104841

**Published:** 2022-02-16

**Authors:** Shulan Mao, Zeyu Shen, Weidong Zhang, Qian Wu, Zhuoya Wang, Yingying Lu

**Affiliations:** ^1^ State Key Laboratory of Chemical Engineering Institute of Pharmaceutical Engineering College of Chemical and Biological Engineering Zhejiang University Hangzhou 310027 China; ^2^ ZJU‐Hangzhou Global Scientific and Technological Innovation Center Hangzhou 311215 China

**Keywords:** high voltage, LiCoO_2_ cathode, lithium concentration gradient, lithium‐ion batteries, outside‐in Li^+^ channel, surface protection

## Abstract

The energy density of batteries with lithium cobalt oxide (LCO) can be maximized by increasing the cut‐off voltage to approach the theoretical capacity limit. However, it is not realized in the practical applications due to the restricted cycle life caused by vulnerable cathode surface in deep delithiation state, where severe side reactions, oxygen/cobalt loss and structure degradation often happen. Here, an outside‐in oriented nanostructure on LiCoO_2_ crystals is fabricated. The outer electrochemically stable LiF and Li_2_CoTi_3_O_8_ particles perform as physical barrier to prevent damage of both cathodes and electrolytes, while the inner F doping promote Li ions diffusivity and stabilize the lattice oxygen. With the spinel‐like transition layer between them, a solid and complete lithium‐ion transport channel generation along the lithium concentration gradient. Under the protection from this structure, the LiCoO_2_ withstand the high voltage of 4.6 V and the LCO/graphite pouch full cell with high loading density exhibits 81.52% energy density retention after 135 cycles at 4.5 V.

## Graphical abstract

To chase the long lifespan of LiCoO_2_ at 4.6 V, an outside‐in oriented nanostructure is fabricated on LiCoO_2_ crystals consisting of the outer electrochemically stable LiF and Li_2_CoTi_3_O_8_ particles, the inner F doping and the spinel‐like transition layer between them. A solid and complete lithium‐ion transport channel is generated along the lithium concentration gradient, keeping the integrity of LiCoO_2_ structure.

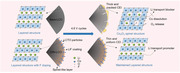

## 1 Introduction

Lithium‐ion batteries (LIBs) have been regarded as the most commercially successful energy storage devices, extensively used in portable electronics, electric vehicles, and grid energy storages.^[^ ^1^, ^2^, ^3^ ^]^ The last 30 years have witnessed the increase of its energy density from 80 Wh kg^−1^ (200 Wh L^−1^) to more than 300 Wh kg^−1^ (700 Wh L^−1^),^[^ ^4^ ^]^ but still cannot satisfy the urgent demands of modern life. The theoretical energy density of intercalation LIBs is directed basically by the ability of cathodes for accommodating Li^+^. Among those cathodes available on the market, LiCoO_2_ (LCO), first commercialized in 1991 by Sony, is still the mainstream cathode in 3C devices due to its excellent conductivity and superior high volumetric energy density.^[^ ^5^ ^]^ Moreover, what attracts people most is that when enhance the cut‐off voltage to 4.6 V versus Li/Li^+^, LCO can release the capacity of ≈220 mAh g^−1^ which gives a ≈30% increase of energy density, comparing the 4.45 V versus Li/Li^+^ (≈173 mAh g^−1^) practically used at present.^[^ ^5^, ^6^, ^7^ ^]^ Maintaining the long‐term stable cycling of LCO under high voltage is the prominent problem for both academia and industry.

As one of the degradation mechanisms, phase transition from O3 to H1‐3 (hybridized O1 and O3 phases) at 4.55 V^[^ ^8^, ^9^ ^]^ can be suppressed by doping with single element^[^ ^10^ ^]^ or multi‐elements.^[^ ^7^, ^11^ ^]^ Although the consensus still does not reach, recent researches emphasized the severity of the surface problems^[^ ^12^, ^13^ ^]^ because the interface is more vulnerable compared with LCO bulk under high voltage. Essentially, high cut‐off voltage means the deep delithiation state of cathodes.^[^ ^3^ ^]^ Lithium in the vicinity of surface is more deficient than inside after charging because of the lithium concentration gradient^[^ ^14^, ^15^, ^16^ ^]^ (Figure S1, Supporting Information). In this condition, oxygen redox (O^2−^→O^*α*−^, *α* < 2) participates in charge compensation, due to the overlap between Co 3d band and O 2p band (strong covalence of Co–O band), leading to oxygen loss and irreversible phase transformation (CoO_2_ →Co_3_O_4_) from the surface of LCO.^[^ ^17^, ^18^ ^]^ Furthermore, O and Co with high oxidation state could react with interfacial electrolytes and generate undesired cathode‐electrolyte interphase (CEI) with consumption of active lithium and cobalt ions.^[^ ^18^ ^]^ It is evidenced that structural collapse and oxygen vacancies originated from surface into interior.^[^ ^12^, ^19^ ^]^ Due to CoO_6_ plane is the skeleton of LCO layered structure, O and Co loss irreversibly destroy the structural integrity. As a result, the increased contact area with electrolyte will intensify the surface structure degradation and interphase side reactions, retarding the Li‐ion diffusion significantly,^[^ ^20^ ^]^ which in turn aggravate crystal fragmentation, generating a vicious circle.

In order to protect LCO from above surface problems so as to realize stable cycling at high‐voltages, a reasonable designed surface coating structure with following features is needed: fast Li^+^ conductor to mitigate the lithium concentration gradient, solid barrier to isolate LCO from electrolytes and lattice stabilizer to inhibit O loss. Traditional surface coating with oxides,^[^ ^21^ ^]^ phosphates,^[^ ^22^ ^]^ and fluorides^[^ ^23^ ^]^ only serves as physical barriers in usual, many of these materials retard the Li^+^ transportation, which is detrimental to ease the lithium concentration gradient. Electrode materials such as LiMn_1.5_Ni_0.5_O_4_ can solve this problem and result in good cycling performance,^[^ ^17^ ^]^ but inevitably lead to dissolution of transition metal ions. In recent years, many works introduced transition layer or solid solution layer into surface protection of LCO^[^ ^24^, ^25^, ^26^, ^27^ ^]^ because this solid conformal structure is able to restrain the phase transition,^[^ ^24^ ^]^ prevent lattice mismatch,^[^ ^25^ ^]^ and inhibit interfacial side reactions.^[^ ^26^ ^]^ For instance, Li–Al–F surface‐modified LiCoO_2_ with Li–Al–Co–O–F surface doping layer restrained the harmful irreversible phase transition at above 4.55 V.^[^ ^24^ ^]^ In addition, spinel phases and Li_3_PO_4_ were formed by reaction between LCO and Li_1.5_Al_0.5_Ti_1.5_(PO4)_3_, which benefit 4.6 V cycle performance at both room temperature and 45 °C.^[^ ^26^ ^]^ However, these strategies did not meet the three requirements simultaneously. More regrettably, they did not carry out a pouch full cell test under high voltages (≥4.5 V) which is urgently required in the industry.

In this work, we fabricated an outside‐in oriented nanostructure on surface of LCO single crystal (OIN‐LCO) to provide multiple protection following the principles of electrochemical stabilizer, Li promoter, and O loss inhibiter. As illustrated in Figure S2, Supporting Information, the OIN‐LCO consists three functional surface layers: 1) Li_2_CoTi_3_O_8_ particles embedded in ultrathin (2–3 nm) LiF coating layer serves as physical isolation from electrolytes especially HF, preventing interfacial side reactions with the dissolution of Co ions and infinite growth of CEI. Li_2_CoTi_3_O_8_ has high lithium‐ion diffusion coefficients^[^ ^28^ ^]^ and the thin LiF coating layer is electrochemical stable.^[^ ^29^ ^]^ 2) Spinel‐like transition phase ≈20–30 nm with trace doping of Ti and F works as solid structure to bridge the Li migration gap between bulk and surface as well as prevent lattice mismatch. 3) Gradient F doping layer with ≈100 nm beneath the coating layer to bulk strongly stabilizes the lattice O and prevents irreversible phase transition to Li‐retarded spinel. The F doping facilitates Li diffusion during deep delithiation state, demonstrated by density functional theory (DFT) calculation. Remarkably, with all these benefits, OIN‐LCO exhibits 81.2% capacity retention after 200 cycles at 4.6 V in half cell and 81.52% energy density retention after 135 cycles at 4.5 V with high loading (13.0 mg cm^−2^) in pouch cell matched with graphite.

## 2 Results and Discussion

### 2.1 Characterization of OIN‐LCO

**Figure 1:**
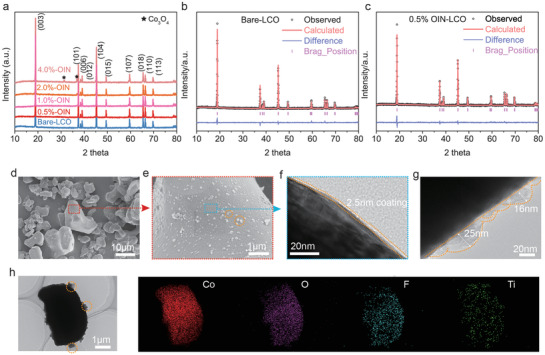
Structure and surface morphology of OIN‐LCO. a) XRD patterns of Bare LCO and 0.5–4.0% OIN‐LCO. b,c) Rietveld refinements of the XRD patterns for Bare LCO and OIN‐LCO. SEM images of OIN‐LCO at d) low and b) high magnification, respectively. TEM images of f) OIN‐LCO at flat area in blue and g) particles aggregation area. h) TEM image and EDS mapping (Co, O, F, Ti) of OIN‐LCO.

This OIN‐LCO was synthesized from Bare‐LCO by solvothermal method, followed by calcination under argon atmosphere, which can be found in Figure S2, Supporting Information, and Experimental Section for detail. To select the optimum coating thickness, we synthesized 0.5%, 1.0%, 2.0%, and 4.0% OIN‐LCO with 0.5–4.0 wt% mass loading of total Li, Ti, F elements, respectively. From the X‐ray diffraction (XRD) pattern and Rietveld refinements (**Figure** **1**a–c), we found that modified OIN‐LCO has same R‐3m layered structure with Bare‐LCO, indicating this surface treatment does not ruin bulk crystalline structure of LCO. But when total coating amounts increase, Co_3_O_4_ (2*θ* = 31.2, 36.8) comes up due to the reaction between LCO and TiO_2_ under high temperature.^[^ ^28^ ^]^ Rietveld refinements results in Figure S3 and Table S1, Supporting Information, show the proportion of Co_3_O_4_ with different coating amounts. As Co_3_O_4_ is considered as “bad spinel” for poor Li^+^ transportation,^[^ ^17^, ^18^ ^]^ 0.5% coating seems appropriate with minimal Co_3_O_4_. This is coincident with scanning electron microscope (SEM) analysis in Figure 1d,e and Figure S4, Supporting Information, in which particles are uniformly distributed on the surface of 0.5% OIN‐LCO while regionally distributed and even aggregated together on the surface of other OIN‐LCO. Therefore, 0.5% OIN‐LCO was chosen for the following characterization. Without specific instructions, OIN‐LCO refers to 0.5% OIN‐LCO.

The inductively coupled plasma mass spectroscopy (ICP‐MS) and ion chromatography results in Figure S5 and Table S2, Supporting Information, evidence that the actual added Li, Ti, F elemental composition is close to the intended (0.5 wt%) with little decrease after annealing. To further identify the OIN‐LCO structure in detail, transmission electron microscope (TEM) was carried out (Figure 1f–h). The coating thickness is ultrathin with ≈2.5 nm, and the grain size varies from 10 to 25 nm. Energy‐dispersive X‐ray spectroscopy (EDS) mapping illustrates the uniform distribution of F whereas Ti is dotted on the matrixes, suggesting continuous F‐enriched coating and scattered Ti‐enriched particles. Using X‐ray photon spectroscopy (XPS, Figure S6, Supporting Information), the surface‐sensitive technique, we found that OIN‐LCO shows strong LiF signal ≈685.0 eV,^[^ ^24^ ^]^ Ti 2p signal at 458.4 and 464.1 eV (absent in Bare‐LCO).^[^ ^30^ ^]^ The peak at 529.9 eV in O1s refers to the Ti—O bonds, according with the Ti‐containing oxides on the surface.^[^ ^28^ ^]^ The explicit chemical components are detected by XRD analysis for the reaction products of LCO and coating materials (1:1 in wt%) after the same synthesis process. Quantitative Rietveld refinement results are exhibited in Figure S7 and Table S3, Supporting Information, suggesting the existence of Li_2_CoTi_3_O_8_, Co_3_O_4_, and LiF. It is important to note that this is not the most accurate method and the amounts of products may vary (very little Co_3_O_4_ in OIN‐LCO), but the presence of these phases is credible.^[^ ^26^ ^]^

**Figure 2:**
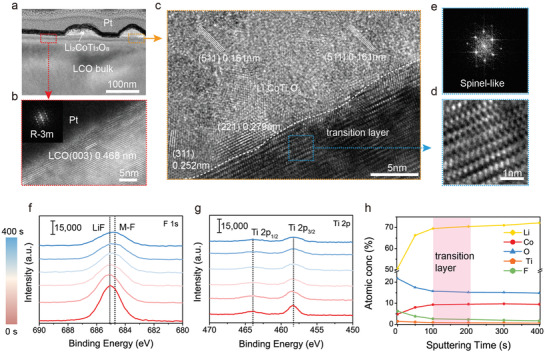
Transition layer and outside‐in element distribution of OIN‐LCO. a) High magnified TEM of OIN‐LCO after FIB treatment. b,c) HRTEM images of selected regions in (a). d,e) Magnified HRTEM image of selected region in c and corresponding FFT pattern. f,g) XPS depth profiles of F 1s, Ti 2p with Ar sputtering of OIN‐LCO. h) Element atomic concentrations of Ti, F, Li, Co, and O in OIN‐LCO from surface to interior obtained from XPS results.

The structure of subsurface is illustrated by focused ion beam (FIB) treatment and high‐resolution TEM (HRTEM, **Figure** **2**a–d). In the region of particles (Figure 2c), lattices spacings of 0.161, 0.252, and 0.279 nm further confirm the crystal structure of Li_2_CoTi_3_O_8_, corresponding to (511), (311), and (221) planes. More excitingly, the spinel‐like transition layer that acts as a link between surface coating and bulk is formed (Figure 2d,e), probably through trace doping of Ti and F, which not only avoids lattice mismatch but also facilitates lithium‐ion transportation from outside to inside.^[^ ^25^, ^26^ ^]^ Further EDS line scan in Figure S10, Supporting Information, verifies the co‐exist of Ti and F elements in this transition layer ≈20–30 nm, beneath which is the F doping region.

In order to confirm the gradient doing of F from surface into interior, XPS etching (Figure 2f–h and Figure S11, Supporting Information) was conducted by increasing the Ar sputtering time to 400 s (≈80 nm). Figure 2h exhibits the functions of atomic concentration (Li, Co, O, Ti, and F) and sputtering time. As etching time increases, the intensity of F 1s signal decreases significantly until 100 s and then drops slowly with following time, accompanying with the appearance of new signal at ≈684.8 eV (indexed into Co—F, Figure 2f),^[^ ^31^ ^]^ which indicates that fluorine element is gradient doped into the crystal lattice beneath transition layer. Meanwhile, the intensity of Ti 2p also becomes weaker as sputtering and almost disappears after 200 s (Figure 2g,h and Figure S11d, Supporting Information), indicating that Ti tends to be in the surface transition layer rather than get into the bulk as easily as F. This is according well with the previous analysis that F is intercalated into lattice while Ti is intended to stay on the surface rather than diffusing inward into the deep bulk interior.^[^ ^7^ ^]^ Fluctuation of Li, Co, O elements in Figure 2h and Figure S11, Supporting Information, during the first 100 s results from the existence of Li_2_CoTi_3_O_8_ particles and LiF coating layer, since XPS technique tests statistical characteristics.^[^ ^32^ ^]^

All these evidences above supported the surface functional structure of OIN‐LCO: the external layer is Li_2_CoTi_3_O_8_ particles embedded in 2.5 nm LiF coating layer; the inside layer is F gradient doping ≈100 nm diffusing into bulk and maintained R‐3m layered structure (Figure 2b); between them is the spinel‐like transition layer with trace Ti and F doping as linkage. This structure forms a lithium‐ion transport channel from the outside to bulk, and is electrochemically stable enough to prevent side reactions and oxygen loss. It is because of this structure that can LCO withstand the severe conditions of high voltages and exhibit enhanced electrochemical performance.

### 2.2 Enhanced Electrochemical Performance

**Figure 3:**
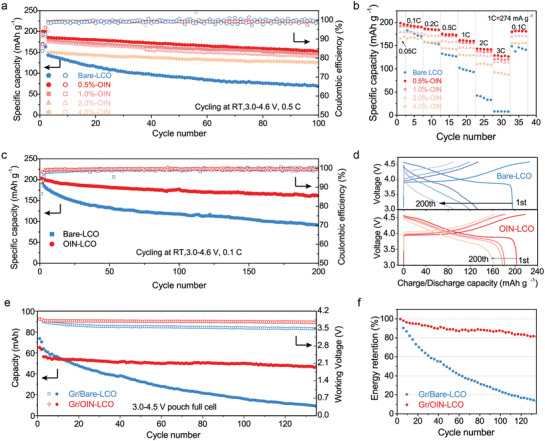
Electrochemical performance of Li/LCO half cells and Graphite (Gr)/LCO pouch full cells with OIN‐LCO versus Bare‐LCO. The cycle performance at a) current density of 0.5 C (1 C = 274 mA g^−1^) and b) corresponding rate performance of Bare‐LCO or OIN‐LCO in half‐cells with different coating weight at room temperature in the voltage range of 3.0–4.6 V (vs Li/Li^+^). c) Long‐term cycling performance of half‐cells with Bare‐LCO or OIN‐LCO electrodes at 0.1 C. d) Discharge–charge profiles of half‐cells with Bare‐LCO or OIN‐LCO electrodes at 1st, 50th, 100th, and 200th cycles at 0.1 C, within 3.0–4.6 V (vs Li/Li^+^). e) The cycling retention of discharge capacities and discharge voltages of Bare‐LCO or OIN‐LCO pouch full cells with graphite anode. f) The energy retention of pouch full cells as a function of cycle number. All cells were pre‐cycled for two cycles at low current density.

The electrochemical performances of half cells and full cells were both evaluated for Bare‐LCO and OIN‐LCO (**Figure** **3** and Figures S12–S15, Supporting Information). First, Figure 3a,b display how the different coating weights of Li, Ti, F elements influence the electrochemical performance at 4.6 V. It is apparent that the OIN‐LCO exceed Bare‐LCO regardless of the coating amount, among which the 0.5% OIN‐LCO shows the best performance of 82.5% retention (153.1 mAh g^−1^) after 100 cycles at 0.5 C (1 C = 274 mA g^−1^, all the retentions are compared with the third cycle as first two cycles are formation process). The capacity retention of 1.0% OIN‐LCO, 2.0% OIN‐LCO, and 4.0% OIN‐LCO are also very good with 81.9%, 79.8%, 84.0%, respectively, but the thicker the coating layer is, the worse the discharge capacity and rate capability perform (Figure 3b). The reason can be retrospect to the formation of Co_3_O_4_ and aggregation of coating particles which impend the Li‐ion transportation. The long‐term cycling performance of coin half cells (Figure 3c,d) are aimed at verifying the cycling stability of 0.5% OIN‐LCO under 4.6 V high voltage. Compared to Bare‐LCO (90.5 mAh g^−1^ after 200th), OIN‐LCO achieves the high discharge capability of 177.4 mAh g^−1^ with 88.8% capacity retention after 100 cycles and discharge capability of 162.2 mAh g^−1^ with 81.2% capacity retention after 200 cycles at the current density of 0.1 C (0.05 C for the first two cycles). The initial Coulombic efficiency of Bare‐LCO is only 86.68%, much lower than OIN‐LCO (93.07%), which increases rapidly to above 99% with average value of 99.57%, stating the side reactions between cathodes and electrolytes are depressed by OIN coating. In Figure 3d, the voltage profile of Bare‐LCO deforms severely after repeated 200 cycles, indicating the serious structural degradation of Bare‐LCO, but OIN‐LCO remains stable.^[^ ^7^, ^10^, ^33^ ^]^

To exploit the potential practical application, we tested the Bare‐LCO and OIN‐LCO in pouch full cells matched with graphite (Gr) anode in room temperature between 3.0 and 4.5 V under 0.2 C after two formation cycles at 0.1 C. The cell configuration can be found in Figure S13 and Table S6, Supporting Information. It can be seen in Figure 3e that the capacity of 5.6 cm × 4.3 cm pouch cells with single layer is almost 60 mAh (13±0.5 mg cm^−2^). The capacity of Bare‐LCO drops dramatically from 63.84 (187.8 mAh g^−1^ in third cycle, Figure S14, Supporting Information) to 9.15 mAh (26.9 mAh g^−1^) after 135 cycles, whereas the OIN‐LCO presents the really enhanced cycling performance from 56.30 to 46.82 mAh (156.1 mAh g^−1^) with 81.52% energy density retention (Figure 3f) after 135 cycles. The discharge average voltage of the OIN‐LCO remains steady of 3.8 V during the cycles but for Bare‐LCO it gradually decreases to 3.49 V. In addition, the more rigorous condition of 4.55 V was expanded in coin type full cell with graphite anode at 0.3 C in Figure S15, Supporting Information. The OIN‐LCO still provides a capacity retention of 76.9% with highly stable working voltage after 100 cycles. This huge difference can attribute to the different surface structure between Bare‐LCO and OIN‐LCO where the Bare‐LCO suffers from the interface side reactions, O/Co irreversible loss and blocked Li transportation in contrast the OIN‐LCO can avoid these problems.

### 2.3 Suppressed O Loss and Promoted Li^+^ Diffusion

**Figure 4:**
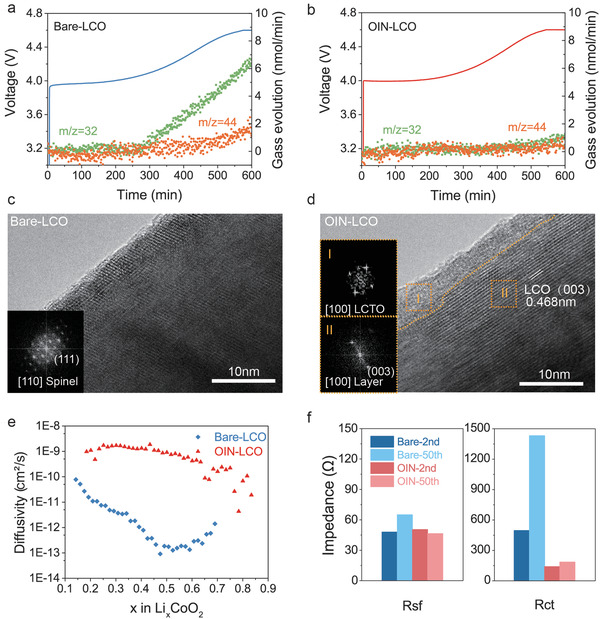
The surface structure evolution and lithium‐ion diffusion kinetics of cathodes during cycling under high voltage. DEMS profiles during the first charging process of a) the Bare‐LCO and b) the OIN‐LCO to 4.6 V. The HRTEM images of c) the Bare‐LCO and d) the OIN‐LCO near the surface after 100 cycles at 0.5 C; insets are the FFT pattern from the selected HRTEM regions. e) The Li^+^ diffusivity within the Bare‐LCO and OIN‐LCO cathodes in the 50th cycles calculated from GITT. f) The impedance of Bare‐LCO and OIN‐LCO resulted from EIS after the cycling of 2nd and 50th at 0.5 C.

The differential electrochemical mass spectroscopy (DEMS) was utilized to monitor the release of O_2_ and CO_2_ in situ during the first charging process. **Figure** **4**a illustrates the emergence of O_2_ and CO_2_ when the Bare‐LCO is charged to ≈4.2 V with rapidly increase in the following charging time. The release of O_2_ indicates that oxygen redox is involved in charge compensation at high voltage, resulting in O loss from surface.^[^ ^17^, ^18^ ^]^ In the same time, the accompanied release of CO_2_ confirms the violent decomposition of electrolytes on the surface of strong oxidizing cathode. However, little O_2_ and CO_2_ are evolved in OIN‐LCO even held in 4.6 V for an hour (Figure 4b), indicating this outside‐in oriented nanostructure protects the LCO surface well. Considering that LiF as the most common solid electrolyte interphase component^[^ ^34^ ^]^ and Li_2_CoTi_3_O_8_ with relatively low electronic conductivity^[^ ^28^ ^]^ both work as physical barrier or HF scavenger, the restriction of oxygen activity can be credited to the F doping which will be explained further with simulation.

The loss of O will lead to the interlaminar densification along with migration of cobalt to the lithium layer to form Co_3_O_4_ spinel structure.^[^ ^14^ ^]^ From the HRTEM and fast Fourier transform (FFT) insets in Figure 4c,d, it is clearly that while the random spinel domains emerge near the surface of Bare‐LCO after 100 cycles, the OIN‐LCO remains well a pristine layered structure with an interplane spacing of 0.468 nm that corresponding to the lattice plane (003) of LiCoO_2_. Besides, spots in the FFT pattern of the region I of OIN‐LCO can be indexed to the surface metallic oxides (Li_2_CoTi_3_O_8_), revealing that the coating layer is stable during cycling. This Co_3_O_4_‐like spinel structure has little storage site for Li and impedes the diffusion of Li^+^, resulting in the capacity degradation and interior resistance increase after repeated cycles.^[^ ^14^, ^17^ ^]^

The galvanostatic intermittent titration technique (GITT) and electrochemical impedance spectroscopy (EIS) were implemented to test this hypothesis. The GITT results in Figure S16, Supporting Information, shows the voltage drop of OIN‐LCO is well restrained in comparison to Bare‐LCO during titrating process in the discharge of 50th cycles. To calculate the Li ion diffusion coefficient (*D*_Li_^+^), we measured the size distribution of two LCO particles with *D*_50_ ≈ 8–9 µm using laser particle analyzer (Figure S17, Supporting Information). Specific formula and calculation process can be found in Table S4, Supporting Information.^[^ ^35^ ^]^ It can be seen in Figure 4e that *D*_Li_^+^ of OIN‐LCO is ≈10^−10^–10^−9^ cm^2^ s^−1^, several orders of magnitude larger than Bare‐LCO. Moreover, charge‐transfer resistance (*R*_ct_) results in EIS (Figure 4f, Figure S18 and Table S5, Supporting Information) also confirm this in which Bare‐LCO has much higher *R*_ct_ than OIN‐LCO after 2 cycles with greatly increase to ≈1400 Ω after 50 cycles. But there is little rise in *R*_ct_ of OIN‐LCO, indicating high Li‐ion diffusivities even after long cycles.^[^ ^36^ ^]^ Besides, the *R*_sf_ represents the surface film (electrolyte/electrode interphase) resistances. After 2 cycles, the *R*_sf_ values of the Bare‐LCO and the OIN‐LCO are similar, but that of Bare‐LCO increases significantly after 50 cycles, demonstrating the thickened CEI at the interface due to electrolyte decomposition. By contrast, *R*_sf_ variation of the OIN‐LCO is not significant, indicating the CEI keeps table after two cycles of formation.^[^ ^11^ ^]^ This will be explained further in next section.

### 2.4 Preventing Surface Side Reactions and Maintained Structural Integrity

**Figure 5:**
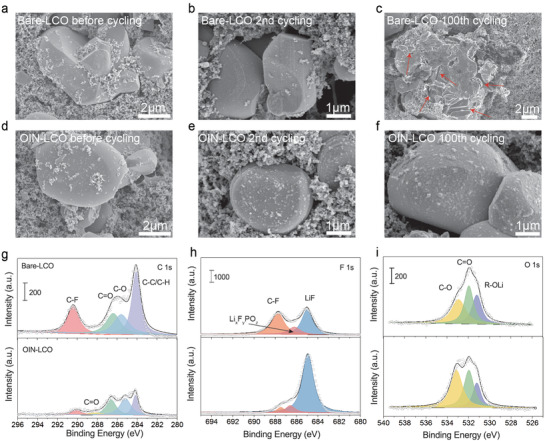
Surface side reactions between cathodes and electrolytes with resulted consequences. a–c) Top‐view SEM images of Bare‐LCO electrodes before cycling and after 2 and 100 cycles under 4.6 V at 0.5 C. d–f) Top‐view SEM images of OIN‐LCO electrodes before cycling and after 2 and 100 cycles under 4.6 V at 0.5 C. XPS analysis of g) C 1s, h) F 1s, and i) O 1s of Bare‐LCO and OIN‐LCO cathodes after 100 cycles under 4.6 V at 0.5 C.

To further illustrate the side reactions of the interface, we performed SEM and XPS characterization on cathodes after long cycles. From the SEM images in **Figure** **5** and Figure S19, Supporting Information, with different magnification, it can be seen that after 2 cycles, a thin film is generated on the exposed surface regions, while that of OIN‐LCO seems to be thicker than Bare‐LCO, which explains why *R*_sf_ of OIN‐LCO is a bit larger. However, after 100 cycles, the very thick CEI ≈22 nm (Figure S20, Supporting Information) and visible micro‐cracks can be found on LCO without protection, in comparison the OIN‐LCO maintains structural integrity and exhibits a similar morphology with the one after 2 cycles. EDS (Figure S21, Supporting Information) and XPS (Figure 5g–i) were carried out to analyze the surface chemistry on the cathodes after 100 cycles. Suffered from the harshly reactive conditions, F and Ti still exist on the surface of OIN‐LCO, reflecting sufficient stability of the fabricated surface structure, while P and F of Bare‐LCO surface come from the decomposition of lithium salt (LiPF_6_). The XPS results further indicate that the main CEI component of OIN‐LCO is LiF (≈685.0 eV) constructed before cycling, rather than the organic products (C═O, C—O, and R‐OLi) resulted from the decomposition of electrolyte solvents which constitute the most parts of CEI for Bare‐LCO.^[^ ^26^, ^37^ ^]^

The side reactions result in electrolytes decomposition and cathode surface degradation simultaneously. ICP technique and EDS mapping were used to demonstrate the relationship between the loss of Co and surface micro‐cracks. The Co content in the electrolyte and graphite anodes after long cycles are both analyzed in Figures S22 and S23, Supporting Information. As expected, the Co loss content from Bare‐LCO is dozens of times larger than OIN‐LCO. The irreversible loss of Co and O will lead to the fracture of the cathode skeleton structure and the deletion of Li active sites, manifesting as the increased polarization and capacity fading (Figures S24 and S25, Supporting Information). Moreover, the (003) peak of Bare‐LCO in XRD pattern (Figure S26, Supporting Information) shifts to the lower degree after 100 cycles at 0.5 C, meaning that the irreversible structure transition gradually accumulates over long cycles, which is possibly due to the loss of Co and O during repeated charge/discharge.^[^ ^10^, ^38^ ^]^ The above analyses indicate that outer layer of LiF and Li_2_CoTi_3_O_8_, as an effective electrochemical inert physical barrier, can inhibit the side reactions on the interface, prevent the dissolution of cobalt and maintain the structural integrity.

### 2.5 Theoretical Understanding of Doping Mechanisms

**Figure 6:**
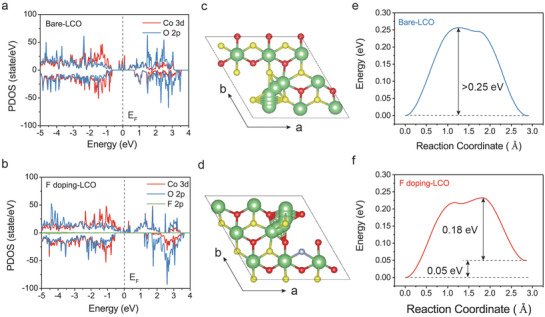
DFT calculations for F substitution. PDOS for a) Li_0.259_CoO_2_ and b) Li_0.259_CoO_1.9815_F_0.0185_. The top view of the Li ions diffusion path for c) Bare‐LCO and d) F‐doping LCO. The green, red, yellow, and purple spheres represent Li, O (below Li), O (over Li), and F atoms, respectively. e,f) The corresponding Li ions migration energy of Bare‐LCO and F doping‐LCO.

In order to understand how F doping affects the oxygen activity and Li^+^ diffusivity, first principles calculations were conducted based on DFT approach. First, the projected electronic density of states (PDOS) of highly delithiated Li_0.259_CoO_2_ and Li_0.259_CoO_1.630_F_0.0158_ (atomic structure can be seen in Figure S27, Supporting Information) are compared in **Figure** **6**a,b. Specifically, the band centers of the O 2p and Co 3d can be seen in Figure S28, Supporting Information, which exhibits the increased energy gap between the O 2p and Co 3d band centers after the F doping (0.7170 eV compared to 0.4572 eV of Bare‐LCO) and the lower O 2p band center position relative to the Fermi level, indicating the weakened oxygen redox activity after F doping.^[^ ^7^, ^10^ ^]^ In addition, Li_0.259_CoO_1.630_F_0.0158_ has much fewer O 2p orbitals than Li_0.259_CoO_2_ at the Fermi level, which supports the experimental DEMS results of the inhibited oxygen loss.^[^ ^19^, ^39^ ^]^ The electronic structure analysis from DFT calculations suggests that the lattice oxygen is stabilized by the F doping strategy, which is consistent well with the XRD Rietveld refinement results. As show in Table S1, Supporting Information, the length of Co—O band becomes longer in the 0.5% OIN‐LCO (1.93215 Å) compared to Bare‐LCO (1.93095 Å), indicating the enhanced ionic characteristic of Co—O bond due to the incorporating of F with strong electro‐negativity which can reduce the proportion of oxygen participating in charge compensation. Next, we simulated the effect of F substitution on the Li ions transportation energy (Figure 6c–f). According to the mechanism of lithium‐ion migration, the lithium atom moves between two of the octahedral sites by passing through the tetrahedral site. So the migration barrier is substantially lower if the intermediate tetrahedral site is surrounded by two vacant octahedral sites as opposed to only one vacant site.^[^ ^14^, ^40^ ^]^ In other words, lithium is more inclined to migrate to the lithium site with a vacancy nearby. Guided by this mechanism, we supposed the path for Li migration is along the diagonal of the plane and there is a Li vacancy next to each destination (Figure 6c,d). F doping‐LCO exhibits the forward Li‐ion migration barrier energy of 0.23 eV and the reverse Li‐ion migration barrier energy of 0.18 eV, less than that for Bare‐LCO of 0.256 eV (Figure 6e,f), indicating F doing facilitates the Li ions transportation. That is, the LCO with F substitution would reduce the barrier energy^[^ ^41^ ^]^ and displayed improved rate performance.

## 3 Conclusions

Based on previous researches, we note that when increase the voltage to 4.6 V to extract more Li form LCO, the gradient of lithium concentration increases. The surface‐initiated O/Co loss accompanying with the irreversible surface structure degradation and infinite growth of CEI would reduce Li storage sites and retard Li^+^ diffusivity, resulting in the endless capacity fading, which are the main obstacles to stable cycling of LiCoO_2_ at high voltage. Considering these daunting challenges on the highly delithiated surface, we constructed an outside‐in oriented nanostructure on LCO crystals, including three functional layers: the Li_2_CoTi_3_O_8_ particles embedded in 2.5 nm LiF coating as the external layer and F gradient doping ≈100 nm diffusing into bulk as the inner layer, between them is the spinel‐like transition layer as linkage. The electrochemically stable LiF and Li_2_CoTi_3_O_8_ perform as physical barrier to prevent damage of both cathodes and electrolytes, while F doping reduces the Li ions migration energy and stabilizes the lattice oxygen. With the transition layer, a solid and complete lithium‐ion transport channel generation along the lithium concentration gradient. As a result, the modified OIN‐LCO exhibits 81.52% energy density retention after 135 cycles on 4.5 V pouch full cell with high loading (13.0 mg cm^−2^), compared to only 13 cycles when Bare‐LCO is used. This work provides an in‐depth analysis between the relationship of LCO structure and performance, which could be extended to other cathodes like lithium‐rich layered materials or Ni‐rich LiNi*_x_* Mn*_y_* Co_1−_ *_x_*_−_ *_y_* O_2_ to approach the theoretical capacity limit for practical applications.

## 4 Experimental Section

### Synthesis

To synthesize the OIN‐LCO, C_16_H_36_O_4_Ti (≥99.0%, Aladdin), LiNO_3_ (99.99%, Aladdin), NH_4_F (99.99%, Aladdin), and LiCoO_2_ (99.5%, Aladdin) were used without further purification as received. Different coating weights (0.5%, 1.0%, 2.0%, 4.0%) OIN‐LCO were synthesized by a facile solvothermal‐assisted method and calcination treatment. The calculated amount of C_16_H_36_O_4_Ti were added in 30 mL absolute ethanol with constant stirring as solution A. Then the calculated amount of LiNO_3_ and NH_4_F were dissolved in 2 mL deionized water as solution B. For example, in the case of forming 0.5% OIN‐LCO, C_16_H_36_O_4_Ti:LiNO_3_:NH_4_F = 0.0114 g:0.0025 g:0.0062 g. Next solution B was added into solution A drop by drop under continuous stirring for 1 h. Then 1 g LCO was mixed with the solution which was further stirred at room temperature for 1 h to obtain a miscible mixture. The mixture was then transferred into a Teflon‐lined autoclave and heated at 180 °C for 5 h for solvothermal reaction. The product was centrifuged and dried in a convection oven. The obtained precursor powder was collected in porcelain boat and calcinated at 700 °C for 6 h under argon atmosphere with a heating rate of 5 °C min^−1^ to get the final product.

### Material Characterizations

The powder XRD patterns were obtained using X‐pert3 Powder (PANalytical B.V., Cu K*α* radiation (*λ* = 1.5406 Å), 40 kV, 40 mA) with a scan range of 10°–80°. The added element composition of coating and Co dissolution content were determined using ICP‐MS (PerkinElmer NexION 300X). The SEM images and corresponding EDS mapping of all materials were obtained using a field emission SEM (HITACHI SU8000) at 5 kV. To access the detailed morphologies of cathode materials, a TEM (HITACHI HT7700) was used at 120 kV and HRTEM was conducted using a cold field emission TEM (JEM‐2100F, JEOL) at 200 kV. A FEI Quanta 3D FEG SEM/FIB Dual Beam was used to prepare the samples. The element analysis of LCO surface before and after cycling was performed by XPS (Thermo ESCALAB 250Xi) with X‐ray excitation source (monochrome Al Ka, power 150 W, X‐ray beam spot 500 µm). The data obtained were corrected by a standard C 1s peak at 284.8 eV. The particle size of LiCoO_2_ was obtained with laser particle analyzer (LS‐230, Coulter). In situ DEMS experiments were performed at Linglu Instruments, Shanghai, using DEMS‐100 with EI of 70 eV as the ion source and secondary electronic multiplier with 1100 V as the detectors. The cell type was Swagelok configuration assembled in glove box. Then the cells were introduced argon for 6 h first and then charged to 4.6 V at 0.1 C.

### Electrochemical Measurements

For half‐cell, the cathode electrodes were fabricated with 80 wt% active material, 10 wt% super P as conductive additive, and 10 wt% polyvinylidene fluoride (PVDF, 99.5%, Arkema) as binder, which was dissolved in N‐methyl‐1,2‐pyrrolidone (NMP, 99.9%, MTI corporation KJ GROUP) and cast onto aluminum foil collector. After NMP solvent was evaporated at 110 °C for 12 h, the electrode was calendared. Coin cells (CR2032) were assembled with the above cathode, lithium metal as anode, polypropylene (PP, Celgard 2400) as separator, and 1 m LiPF_6_ in ethyl carbonate/diethylene carbonate (EC/DEC 1:1 in volume ratio, 50 µL) as electrolyte in an argon‐filled glovebox. The loadings of active cathode materials in all cells were kept 8–9 mg cm^−2^.

For full‐cell, the cathode was composed of 92 wt% active material, 4 wt% super P, and 4 wt% PVDF with the same preparation process as half‐cell. The negative electrode was fabricated with 92 wt% graphite (99.5%, 330 mAh g^−1^, Shanshan Technology Co., Ltd.), 3 wt% super P as conductive additive, and 5 wt% PVDF as binder. The obtained slurry was cast onto copper foil, then it underwent the same process as the cathode. The loading density of LCO cathode was 12.5–13.5 mg cm^−2^ with ≈2.6 mAh cm^−2^ (tested in half‐cell under 0.1C); the graphite anode had a loading density of 8.5–9 mg cm^−2^ with ≈2.9 mAh cm^−2^ (tested in half‐cell under 0.1C). The pouch‐type full cells with a N/P capacity ratio 1.1:1 with dimensions of 5.6 cm × 4.3 cm were assembled using pouch‐cell production machines (MTI corporation KJ GROUP) with single layer of electrodes (one graphite anode foil with single‐side coated and one LCO cathode foil with single‐side coated). The electrolyte was the commercial 1.2 m LiPF_6_ dissolved in a mixture of EC and DEC with a volume ratio of 1:1, with 2 wt% vinylene carbonate as additive.

The galvanostatic charge–discharge measurements were carried out using a Land CT2001A battery test system in a voltage range of 3.0–4.6 V at various C rates at room temperature for the half cells. For the full‐cell tests, a constant current and constant voltage mode was used. The cells were charged at 0.2 C to 4.5 V and then held until the current dropped to 0.1 C. The discharge process was conducted at a constant current mode at 0.2 C. The cells were cycled at the first two cycles for the formation process. EIS, CV, and GITT were tested in an electrochemical workstation (AMETEK) at room temperature. The frequency range of EIS was 100 kHz–0.1 Hz. The CV curves were obtained at a rate of 0.1 mV s^−1^ in a voltage range of 3.0–4.6 V (vs Li/Li^+^). The GITT was tested with constant current for 600 s followed with 3600 s relaxation.

### Simulations

In this work, the well‐resolved Quantum Espresso was used for all of our DFT calculations.^[^ ^42^ ^]^ The projector‐augmented wave was used to describe the ion–electron interaction.^[^ ^43^ ^]^ The exchange–correlation functional was described by generalized gradient approximation of Perdew–Burke–Ernzerhof (GGA‐PBE).^[^ ^44^ ^]^ The Monkhorst–Pack technique was employed to generate k‐point grids for the Brillouin zone sampling.^[^ ^45^ ^]^ Since GGA cannot correctly reproduce the localized electronic states of the transition metal oxide materials, the GGA + U method was used.^[^ ^46^ ^]^ The U values for the Co 3d states were chosen to be 4.9 eV. A 480 eV energy cutoff for the plane‐wave basis set is used to achieve both computational accuracy and efficiency. For geometry optimization, the conjugate‐gradient algorithm was used to relax the ions until the conditions of convergence, 10^−6^ eV per atom in energy and 0.02 eV Å^−1^ in force were reached. Furthermore, the nudge elastic band method was used to evaluate Li‐ion diffusion pathways and the diffusion barrier.^[^ ^47^ ^]^ The unit cells of LiCoO_2_ with F doping were modeled by the experimental results. The 3 × 3 × 1 supercell containing 27 Li atoms, 54 O atoms and 27 Co atoms was adopted in this work. The delithiated‐state Li_0.259_CoO_2_ was modeled by extracting 20 out of 27 Li ions from the LiCoO_2_ slab system. The F‐doped Li_0.259_CoO_1.9815_F_0.0185_ slab system was modeled by substituting 1 F ions out of 54 O ions.

## Conflict of Interest

The authors declare no conflict of interest.

## Supporting information

Supporting Information

## Data Availability

The data that support the findings of this study are available from the corresponding author upon reasonable request.
