# Supplementary material for: Outside‐In Nanostructure Fabricated on LiCoO2 Surface for High‐Voltage Lithium‐Ion Batteries
Source: Adv Sci (Weinh). 2022 Feb 16;9(11):2104841. doi: 10.1002/advs.202104841 (PMC9008786; doi:10.1002/advs.202104841)
Supplement: Supplementary file 1 — Supporting Information [file ADVS-9-2104841-s001.pdf]

ADVANCED SCIENCE
Open Access

Supporting Information

for *Adv. Sci.,* DOI: 10.1002/advs.202104841

Outside-In Nanostructure Fabricated on $LiCoO_2$ Surface for High-Voltage Lithium-Ion Batteries

*Shulan Mao, Zeyu Shen, Weidong Zhang, Qian Wu, Zhuoya Wang, Yingying Lu**

Supplementary information

Outside-In Nanostructure Fabricated on $LiCoO_2$ Surface for High-Voltage Lithium-Ion Batteries

*Shulan Mao, Zeyu Shen, Weidong Zhang, Qian Wu, Zhuoya Wang, Yingying Lu**

S. Mao, Z. Shen, W. Zhang, Q. Wu, Z. Wang, Prof. Y. Lu

State Key Laboratory of Chemical Engineering, Institute of Pharmaceutical Engineering, College of Chemical and Biological Engineering, Zhejiang University, Hangzhou 310027, China

ZJU-Hangzhou Global Scientific and Technological Innovation Center, Hangzhou 311215, China

Email: yingyinglu@zju.edu.cn

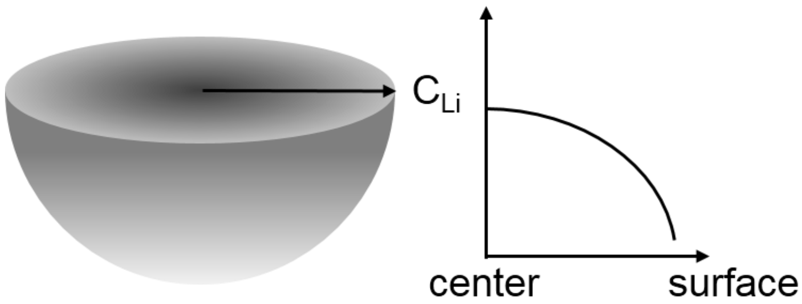

Figure S1. Schematic diagram of Li concentration gradient of LCO single crystal at charging state. Because of transport kinetics, the surface Li concentration is different from center.[1]

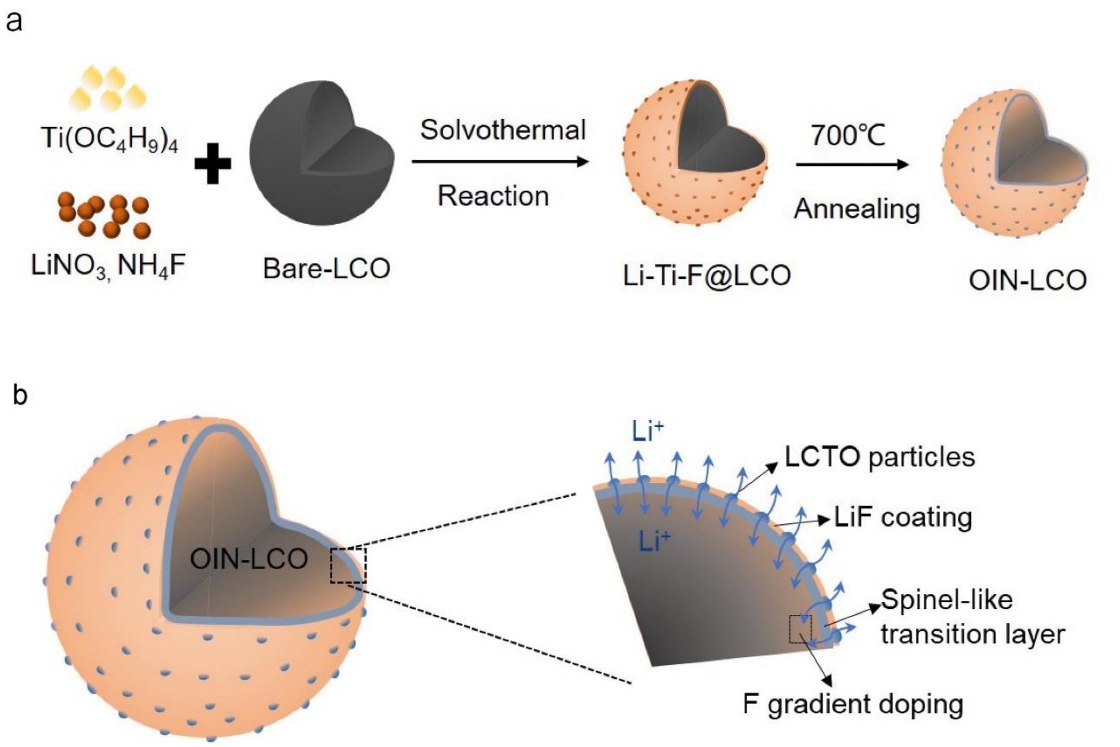

Figure S2. Schematic diagram of the synthesis process and the surface engineering of OIN-LCO. After the solvothermal and annealing treatment, functional layers formation on the surface of LCO.

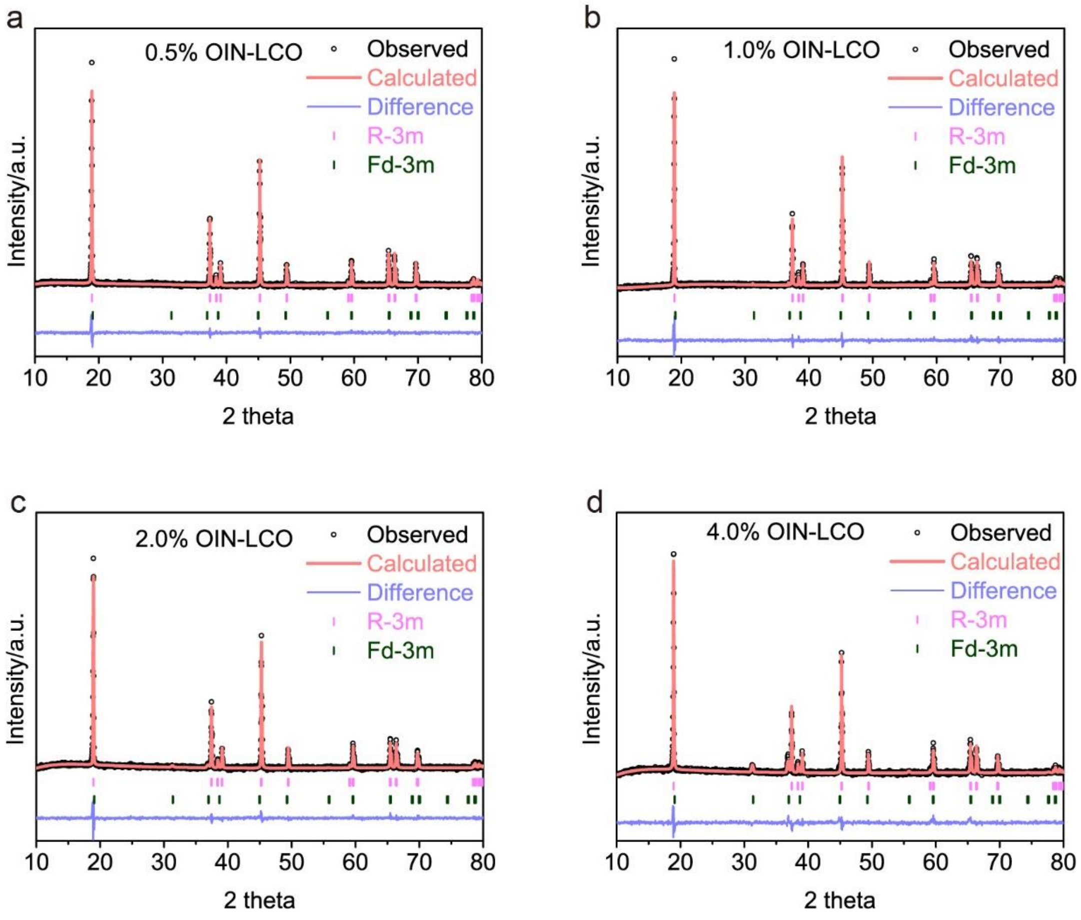

Figure S3. a-d) Rietveld refinements of the XRD patterns for 0.5% - 4.0% OIN-LCO.

R-3m is the space group of LCO and Fd-3m is the space group of Co_3O_4.

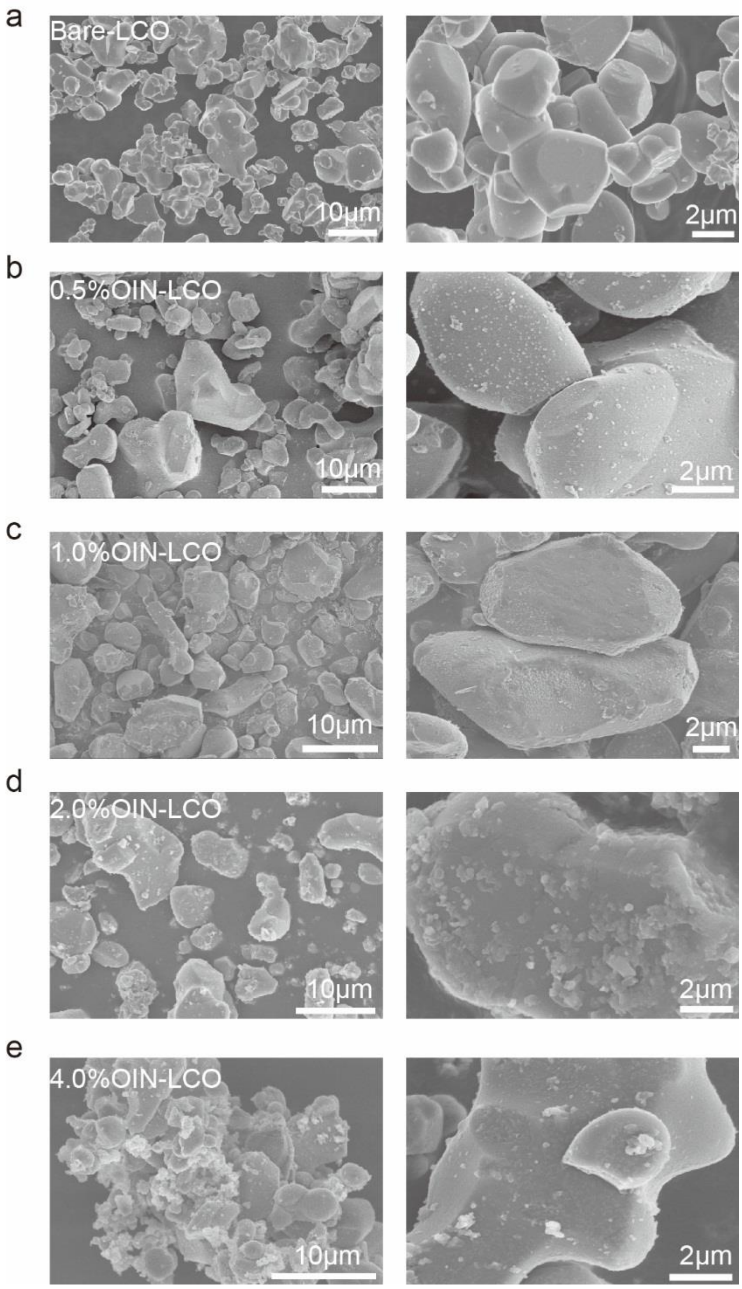

Figure S4. Morphologies of Bare-LCO and OIN-LCO with different coating weight. The SEM images of a) Bare-LCO. b) 0.5%OIN-LCO. c) 1.0%OIN-LCO. d) 2.0%OIN-LCO. e) 4.0%OIN-LCO.

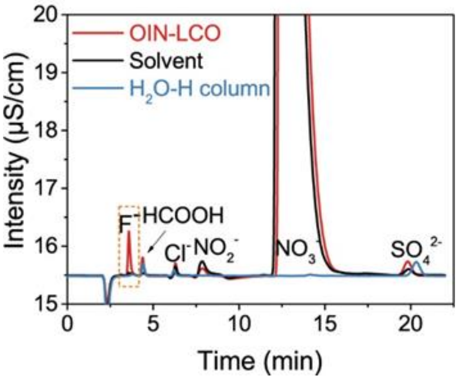

Figure S5. Ion chromatography (IC) of OIN-LCO after dissolution.

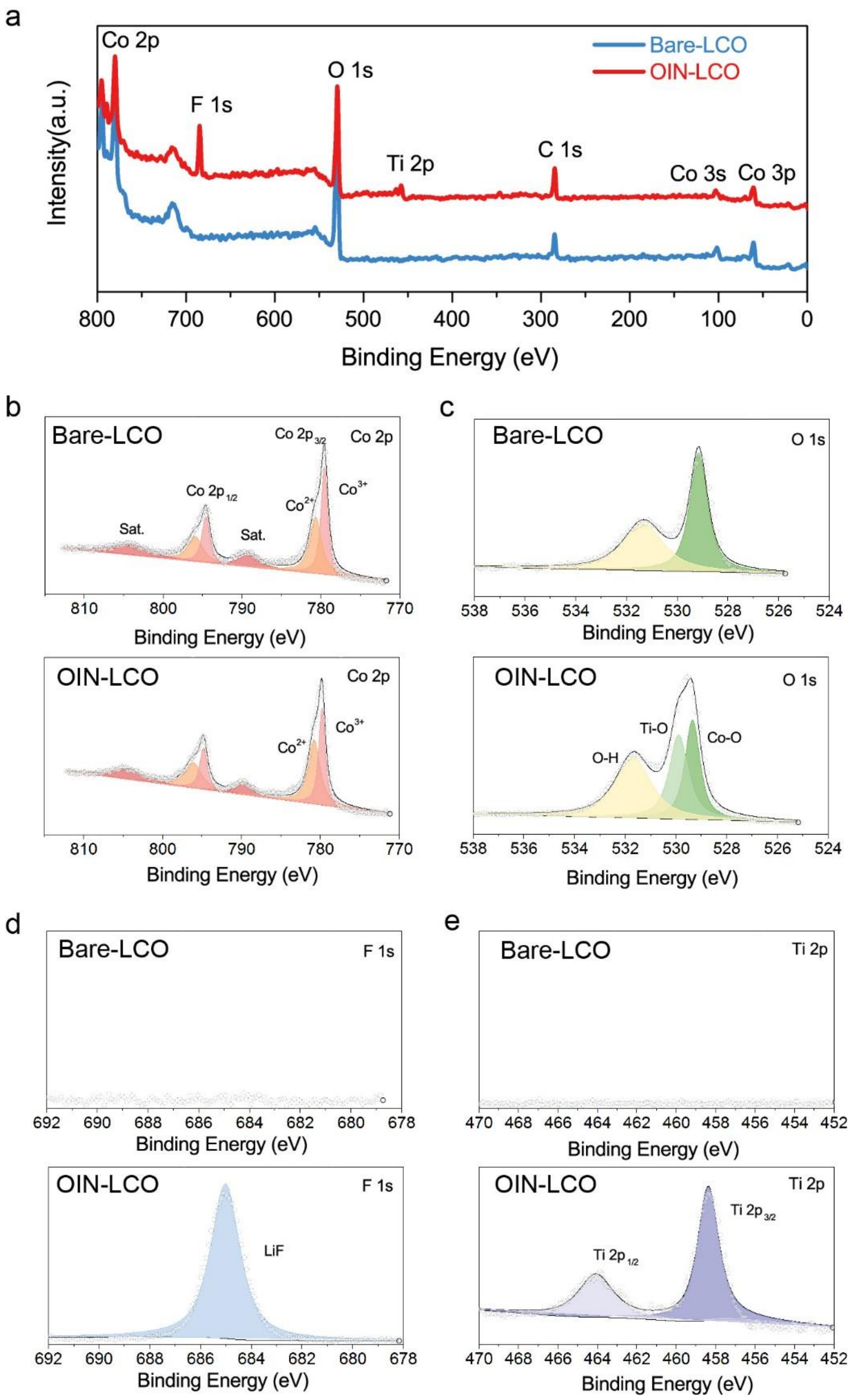

Figure S6. XPS characterization. a) XPS patterns of Bare-LCO and OIN-LCO. b-e) Fine XPS scans of Co 2p peaks, O 1s peaks, and F 1s peaks, Ti 2p peaks respectively.

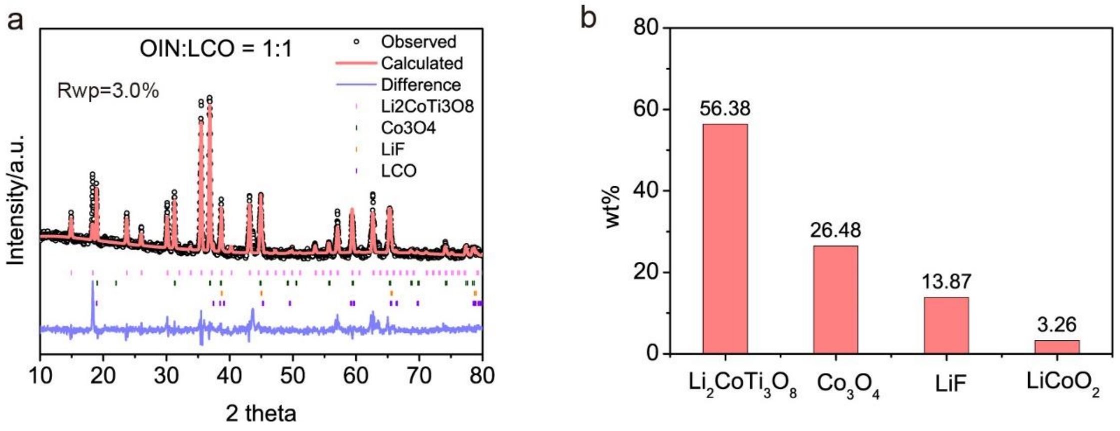

Figure S7. a) Quantitative multiphase Rietveld refinement of reaction products of LCO and coating materials (1:1) after the same solvothermal and calcination process. b) the phases constitution results of Rietveld refinement.

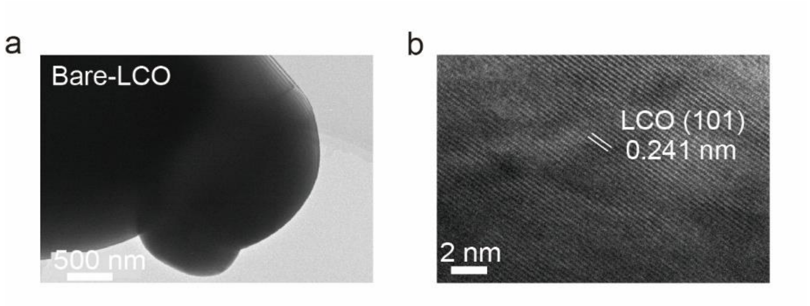

Figure S8. HRTEM images of Bare-LCO.

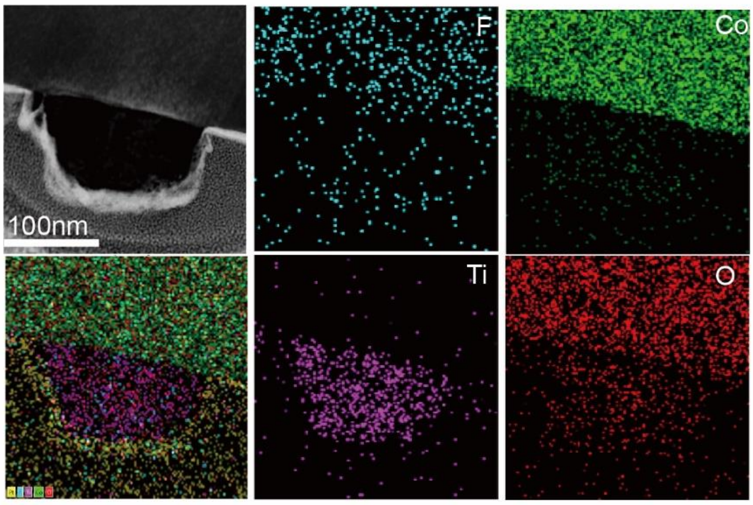

Figure S9. EDS mapping of corresponding TEM images of OIN-LCO from surface to subsurface regions.

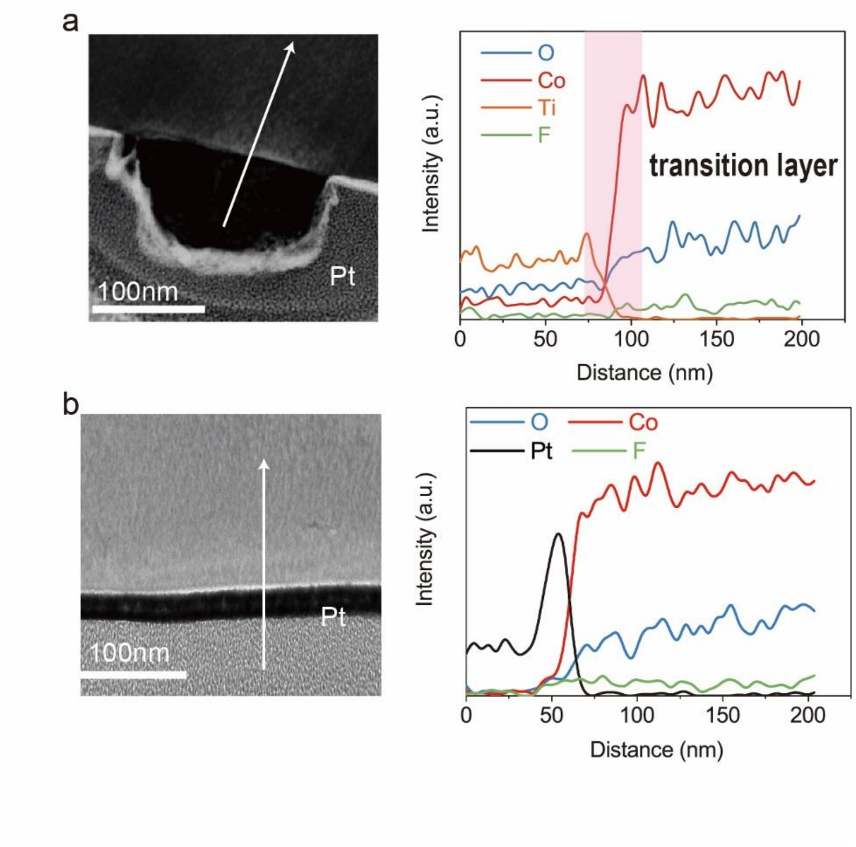

Figure S10. EDS line scanning of corresponding TEM images of OIN-LCO from surface to subsurface regions.

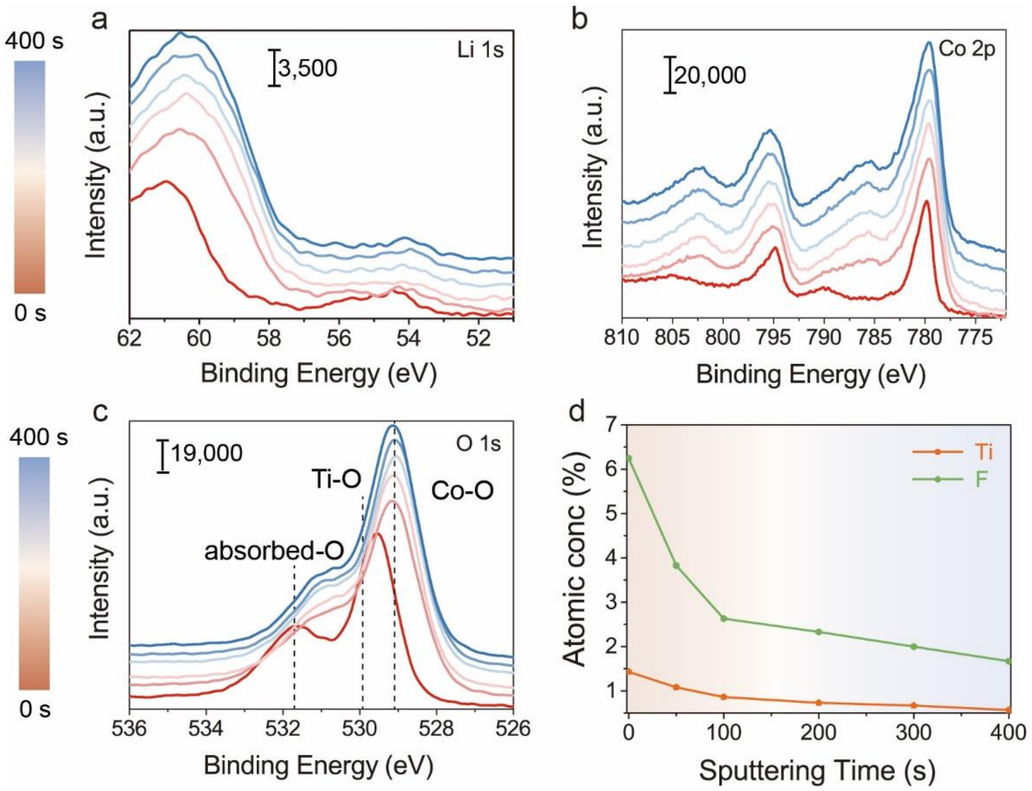

Figure S11. a-c**)** XPS depth profiles of Li 1s, Co 2p, O1s with Ar sputtering of OIN-LCO. d) Enlarged Ti, F distribution in OIN-LCO from surface to interior obtained from XPS results.

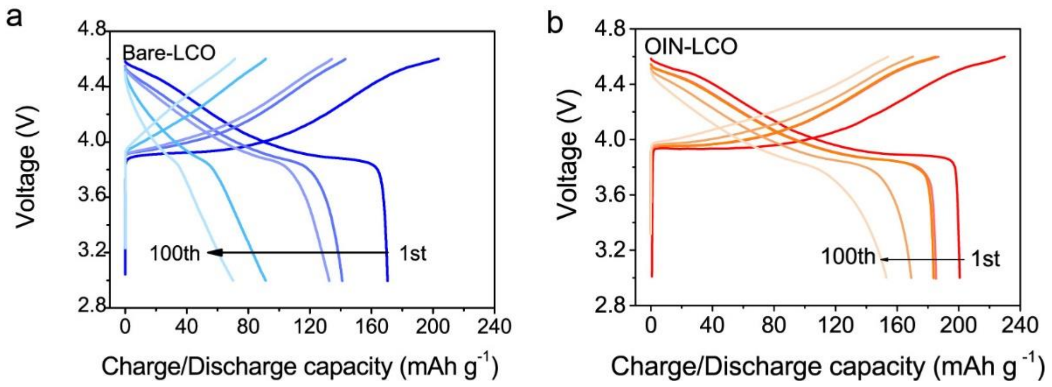

Figure S12. Discharge-charge profiles of half-cells with Bare-LCO or OIN-LCO electrodes at 1st, 5th, 10th, 50th and 100th cycles at 0.5 C within 3.0-4.6 V.

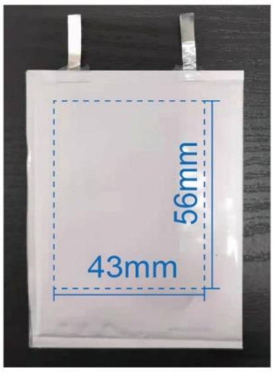

Figure S13. Optical photograph of LCO-Graphite pouch-type full cell.

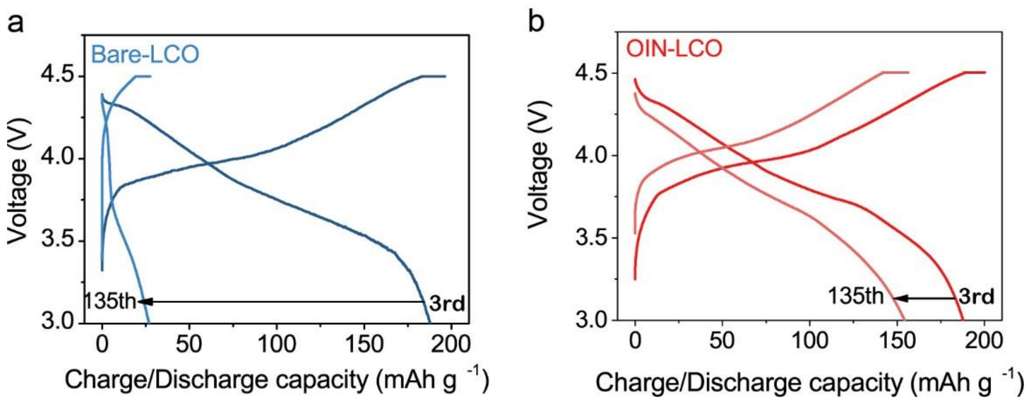

Figure S14. Discharge-charge profiles of full cells with Bare-LCO or OIN-LCO electrodes at 3th and 135th cycles at 0.2 C within 3.0-4.5 V.

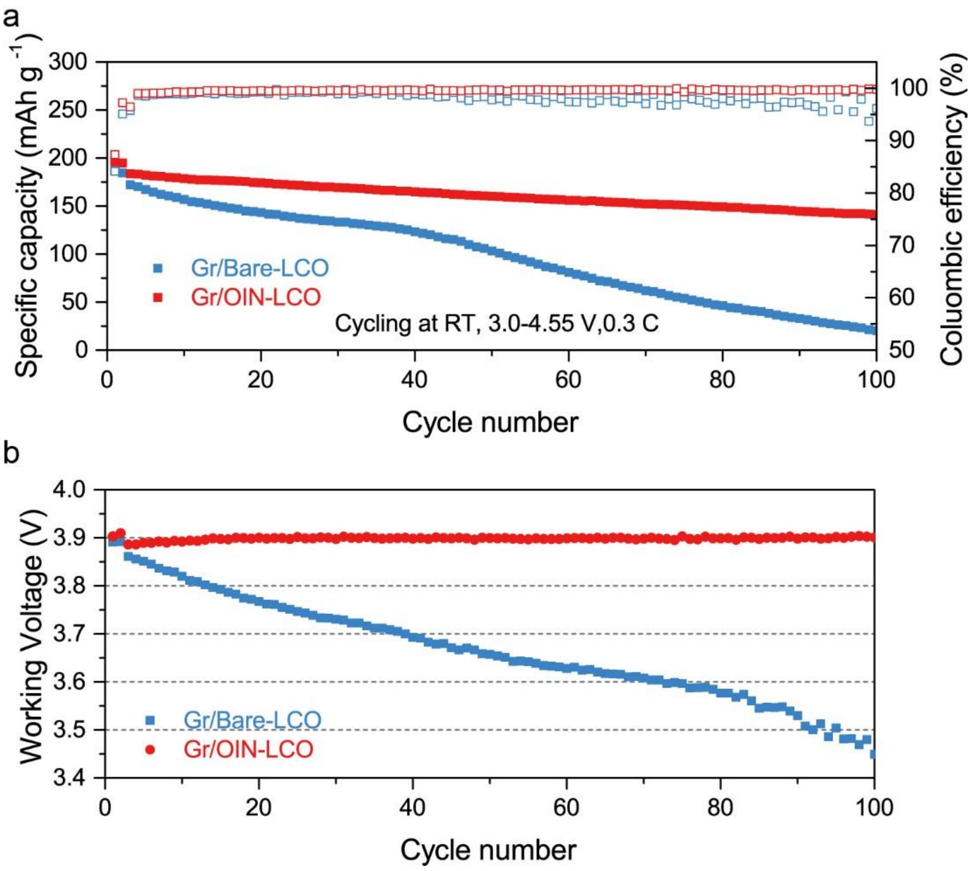

Figure S15. a) Cycle performance of coin full cells with Bare-LCO and OIN-LCO electrodes at room temperature within 3.0–4.55 V (vs. graphite) at 0.3 C. b) Working voltage of full cells with Bare LCO and OIN-LCO electrodes.

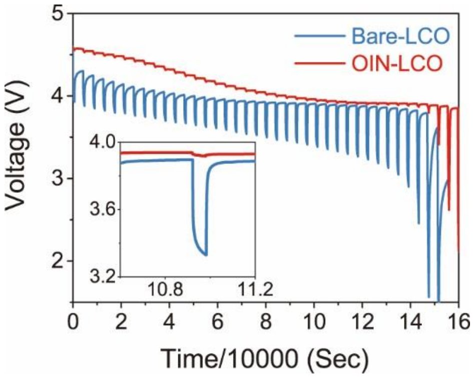

Figure S16. The GITT curve of Bare-LCO and OIN-LCO during discharge in the 50th cycle in the coin half-cells from 4.6 V to 3.0 V.

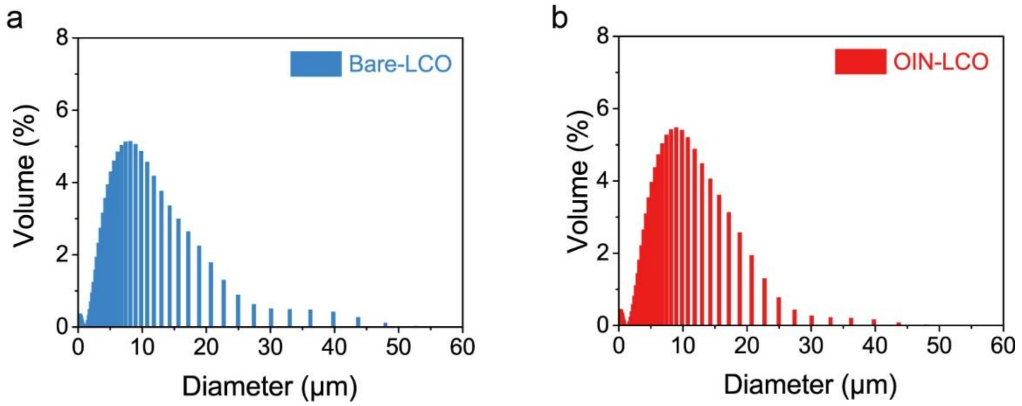

Figure S17. Size distribution of LCO particles: a) Bare-LCO and b) OIN-LCO.

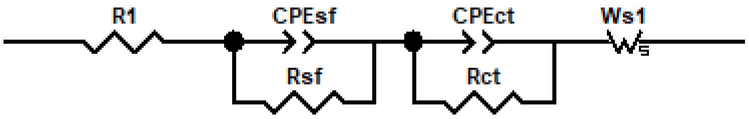

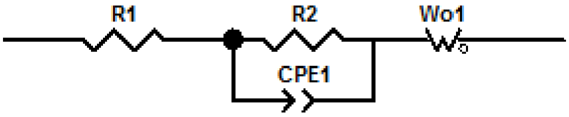

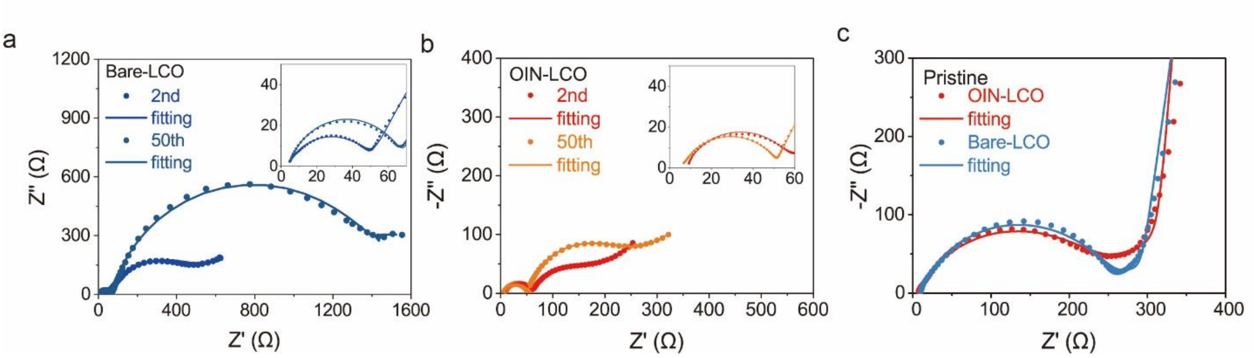

Figure S18. Electrochemical impedance spectroscopy of a) Bare-LCO and b) OIN-LCO fully discharged at 2nd, 50th cycles and c) before cycling.

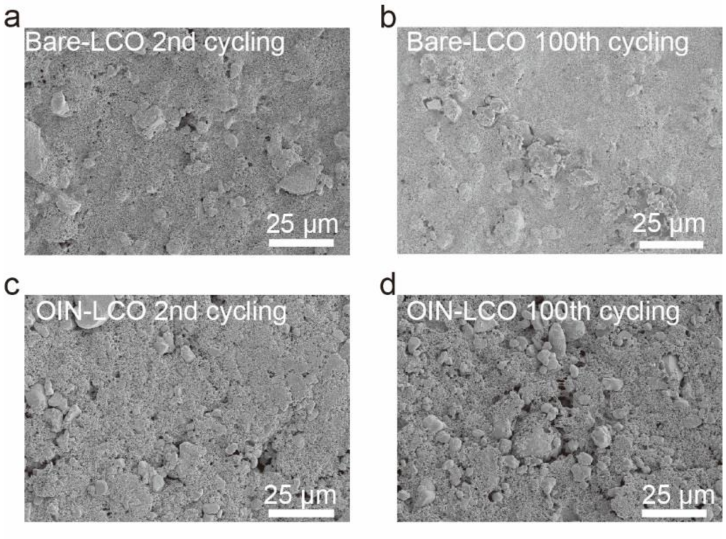

Figure S19. SEM images with low magnification of a,b) Bare-LCO and c,d) OIN-LCO electrodes after 2 and 100 cycles respectively under 4.6 V at 0.5 C.

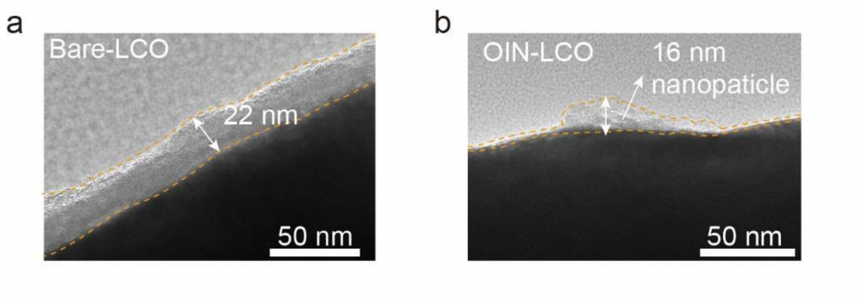

Figure S20. TEM images of a) Bare-LCO and b) OIN-LCO on the surface after 100 cycles under 4.6 V at 0.5 C.

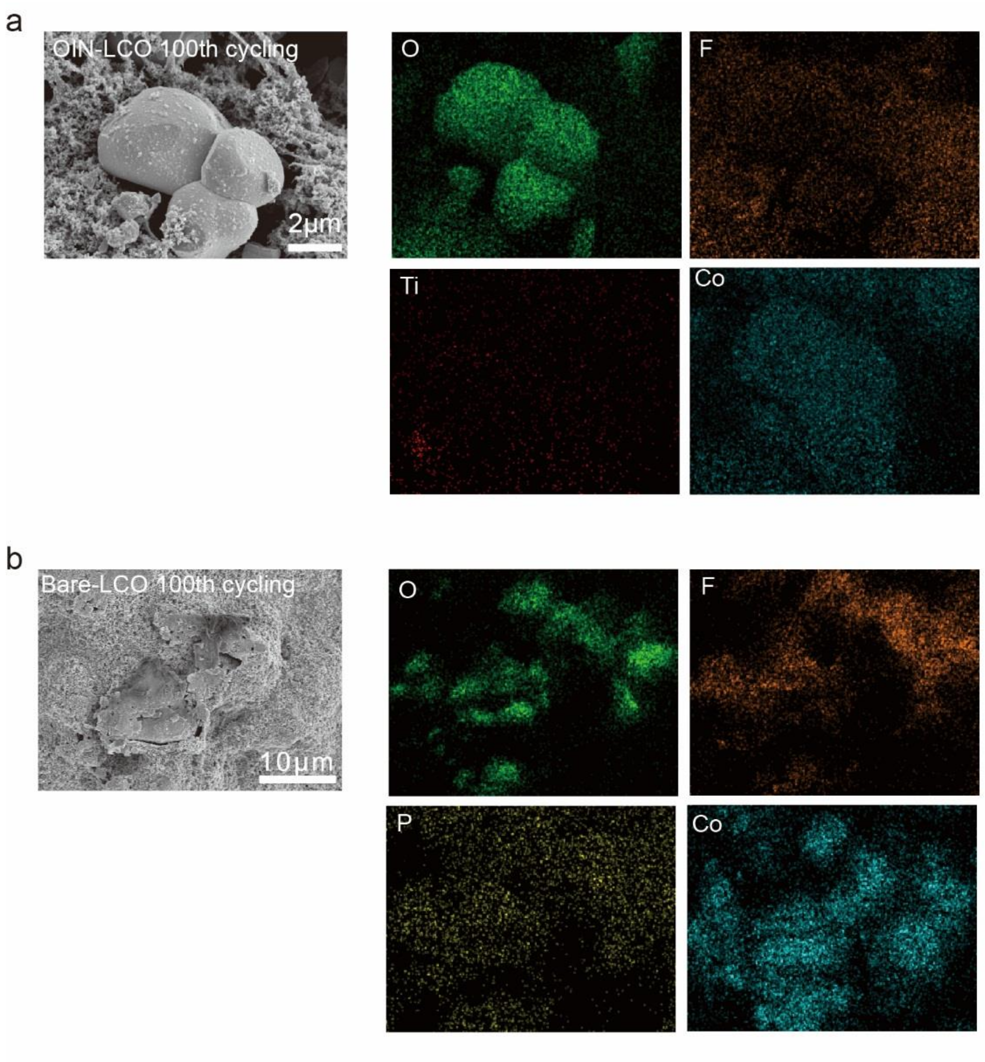

Figure S21. SEM images and EDS mapping of a) OIN-LCO and b) Bare-LCO after 100 cycles under 4.6 V at 0.5 C.

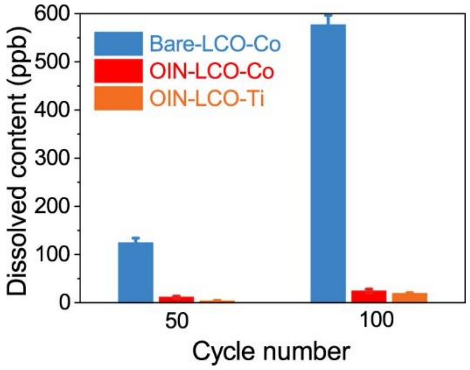

Figure S22. Co content dissolved in the electrolyte of EC:DEC (v:v = 1:1) at different cycles under 4.6 V at 0.5 C.

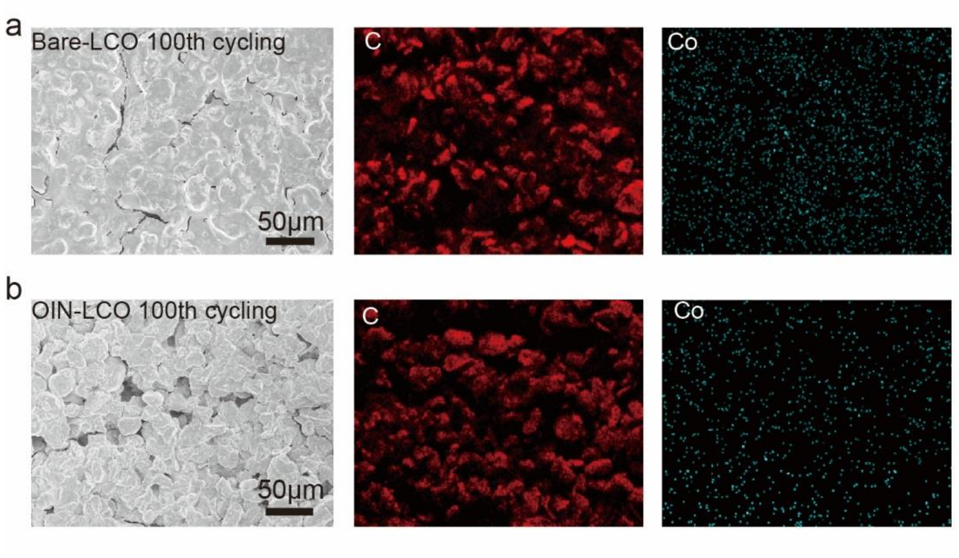

Figure S23. SEM images and corresponding EDS mapping of graphite anodes in full cells of a) Bare-LCO and b) OIN-LCO after 100 cycles under 4.55 V at 0.3 C.

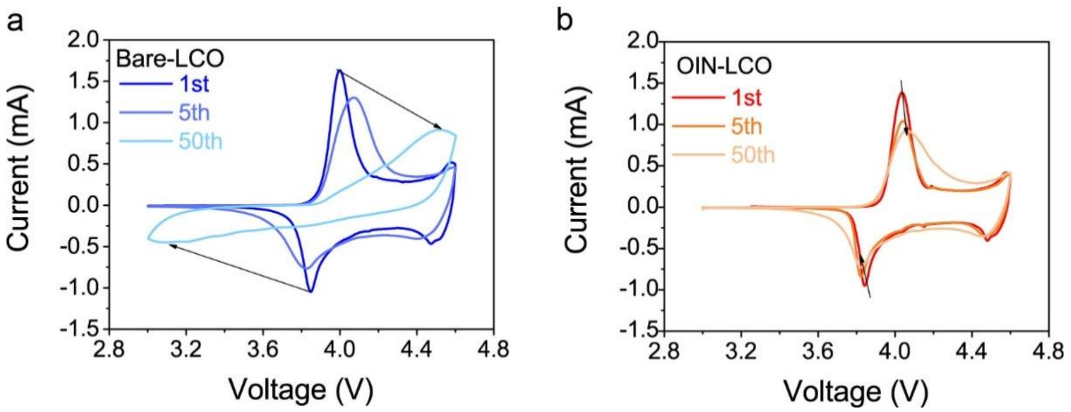

Figure S24. Cyclic voltammograms of half cells with a) Bare-LCO and b) OIN-LCO electrodes at a scan rate of 0.1 mVs^{-1} in the voltage range of 3.0–4.6 V (vs Li/Li^{+}) at 1st, 5th and 50th cycles.

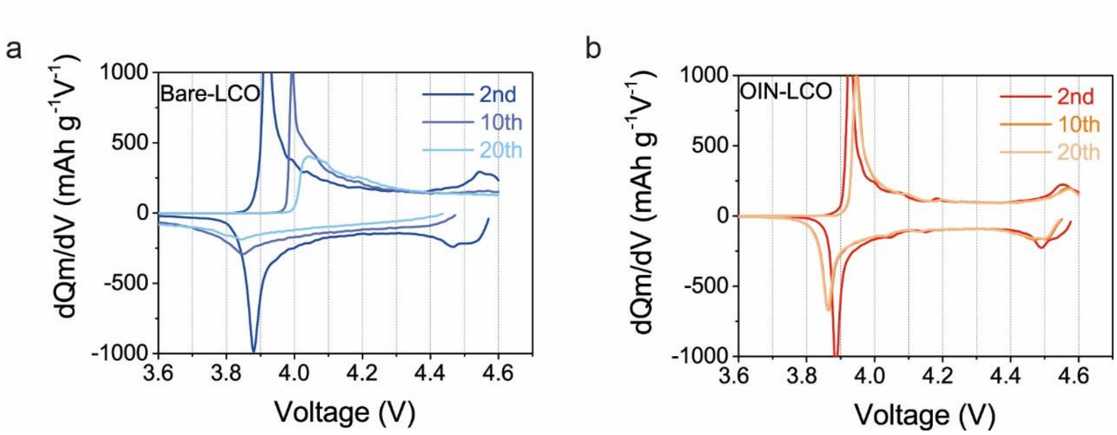

Figure S25. The dQ/dV curves of half cells with a) Bare-LCO and b) OIN-LCO electrodes under 4.6 V at 2nd, 10th and 20th cycles.

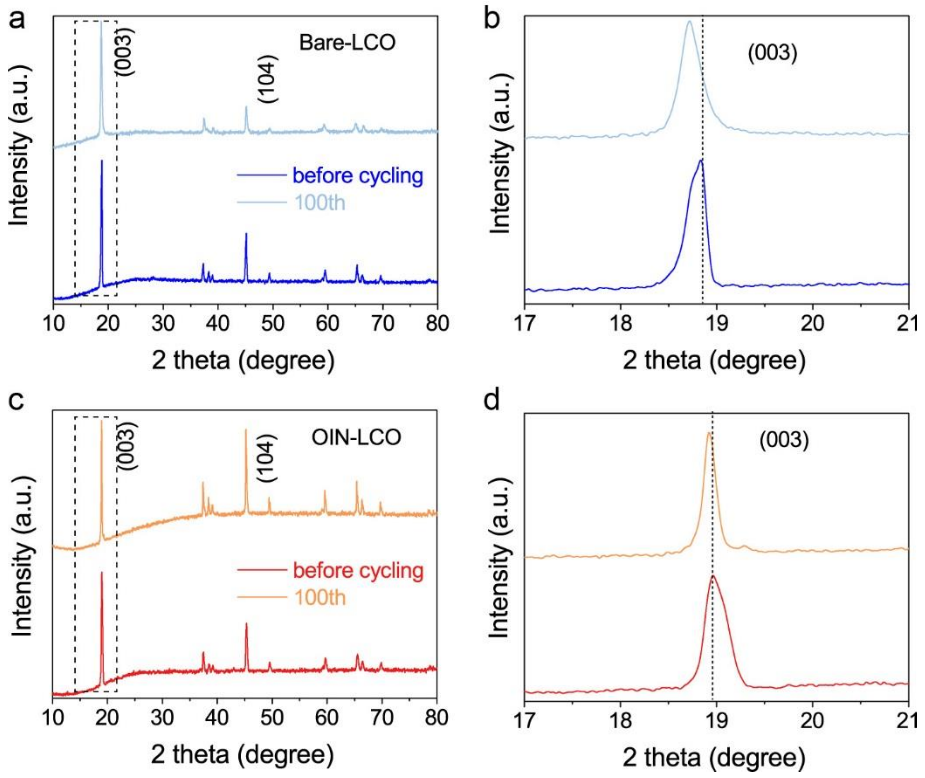

Figure S26. The XRD patterns and (003) peaks evolution of a) Bare-LCO and b) OIN-LCO before cycling and after 100 cycles at 0.5 C.

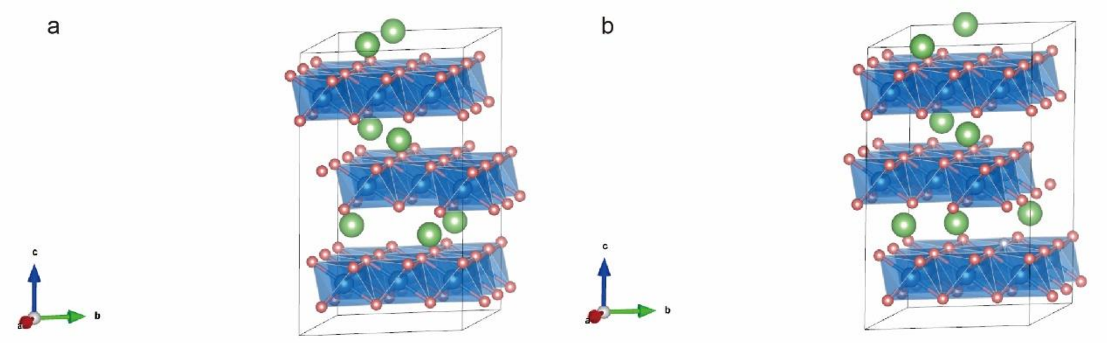

Figure S27. Optimized atomic structure of a) $Li_{0.259}CoO_2$ and b) $Li_{0.259}CoO_{1.630}F_{0.0158}$.

The green, blue, red and gray spheres represent Li, Co, O and F atoms, respectively.

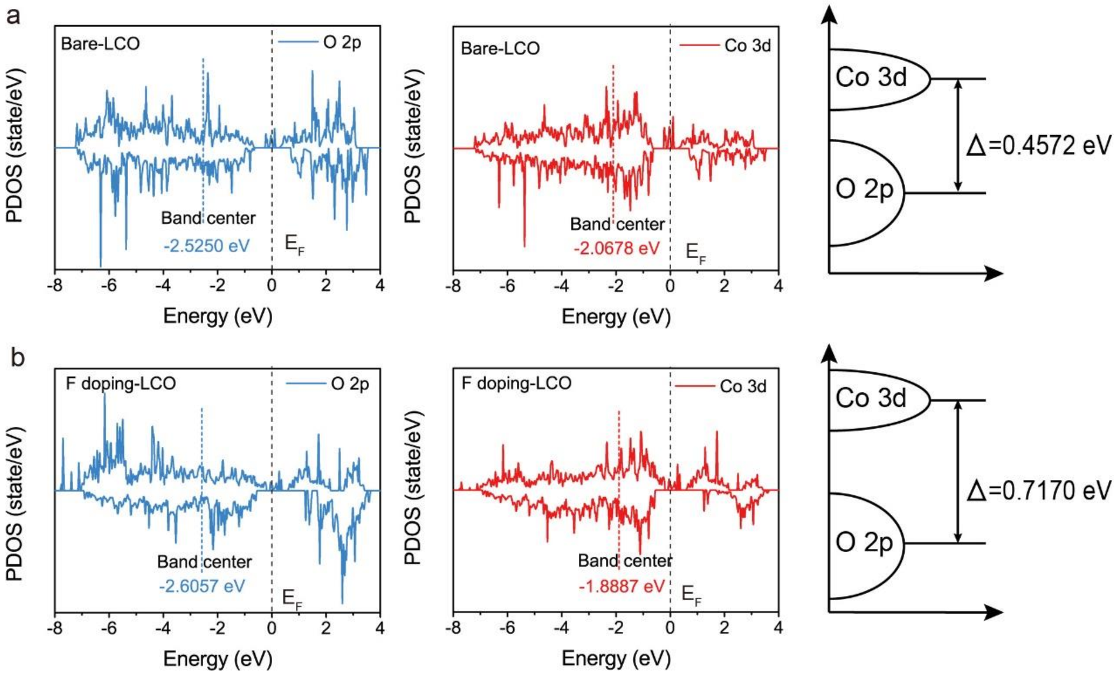

Figure S28. The calculated Projected density of states (the O2p DOS and the Co 3d DOS) for $Li_{0.259}CoO_2$ (a) and $Li_{0.259}CoO_{1.9815}F_{0.0185}$ (b) with the diagrams of the energy gap between Co3d and O2p band center.

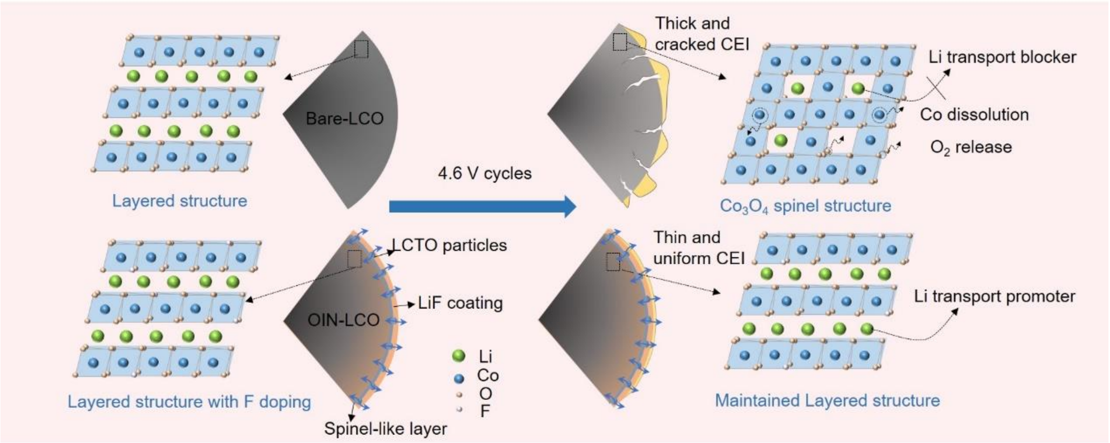

Figure S29. Schematic diagram of different evolution between Bare-LCO and OIN-LCO cycling under high voltage of 4.6 V vs. Li/Li^+.

Table S1. XRD Rietveld refinement results of cell parameters and Fd-3m phase constitution of Bare LCO and 0.5% - 4.0% OIN-LCO.

Samples	a(Å)	c(Å)	V(Å^3)	Fd-3m (wt%)	TM-O	Rwp (%)	Rp (%)
Bare LCO	2.8156	14.0573	96.511	/	1.93095	1.61	1.13
0.5% OIN-LCO	2.8172	14.0676	96.694	0.000745	1.93215	1.94	1.22
1.0% OIN-LCO	2.8173	14.0688	96.707	0.348	1.93224	2.02	1.28
2.0% OIN-LCO	2.8176	14.0700	96.736	1.642	1.93243	1.95	1.23
4.0% OIN-LCO	2.8151	14.0576	96.481	4.847	1.93074	1.98	1.32

Table S2. Chemical composition of Bare-LCO and 0.5%OIN-LCO obtained from ICP-MS and IC.

Sample	Wight percentage /%				Mole ratio over Co/%			
	Li	Co	Ti	F	Li	Co	Ti	F
Bare-LCO	7.474	59.876	/	/	105.977	100.00	/	/
OIN-LCO	7.646	60.882	0.078	0.230	106.631	100.00	0.160	1.191

Table S3. Quantitative multiphase Rietveld refinement results of reaction products of LCO and coating materials (1:1) after the same solvothermal and calcination process.

Phase	Weight ratio(%)	Space group	a(Å)	b(Å)	c(Å)	V(Å^3)
$Li_2CoTi_3O_8$	56.38	P_4332	8.3809	8.3809	8.3809	588.671
Co_3O_4	26.48	Fd-3m	8.0800	8.0800	8.0800	527.512
LiF	13.87	Fm-3m	4.0269	4.0269	4.0269	65.299
$LiCoO_2$	3.26	R-3m	2.8166	2.8166	14.0443	96.488

Table S4. Li ion diffusion coefficient calculation example via GITT technique in 50th discharging stage of Bare-LCO and OIN-LCO.

	x	τ/s	r/cm	$\triangle E_s$/V	$\triangle E_t$/v	D/cm^2 s^{-1}
Bare-LCO	0.308	600	0.81x10^{-3}	0.0152	0.2939	3.73E-12
OIN-LCO	0.301	600	0.8910^{-3}	0.0353	0.035	1.72E-09

The lithium diffusion coefficients for Bare-LCO and OIN-LCO in 50th discharging stage were determined via the GITT technique as shown in the following equation. In the premise of pulse time (τ) is short and the pulse current is small, the lithium-ion diffusion coefficient (D_{Li+}) can be described by Equation:

$$D_{Li^+} = \frac{4r^2}{\pi\tau}\left(\frac{\Delta Es}{\Delta Et}\right)^2$$

where r is the particle size of the cathode materials, ΔE_s is the change of the steady-state voltage of cell for this step, and ΔE_t is the total transient voltage change of the cell for applied current for time τ.[2]

Table S5. Interfacial resistances and charge-transfer resistances of Bare-LCO and OIN-LCO before cycling or fully discharged at 2nd, 50th cycles.

Sample	Cycle number	R_{Sf}	R_{CT}
Bare-LCO	pristine		246.2
	2nd	47.97	496.5
	50th	65.06	1431
LTF-LCO	pristine		238.2
	2nd	50.52	139.7
	50th	46.43	185.4

Table S6. Pouch full-cell parameters.

	Parameter	
	Cathode	Anode
Sample	Bare-LCO / OIN-LCO	Graphite (Gr)
Cell dimension	43 mm x 56 mm	45 mm x 58 mm
Composition	Active material: SP: PVDF = 92: 4: 4	Active material: SP: PVDF = 92: 3: 5
One-side loading (mg cm^{-2})	13.0 ± 0.5	8.8 ± 0.2
Electrode thickness (μm)	38 ± 1	85 ± 1
Current collector thickness (μm)	15 (Al)	10 (Cu)
Electrode density (g cm^{-3})	3.42 ± 0.22	1.04 ± 0.3
Separator thickness (μm)	25	
Negative/Positive ratio (N/P)	1.1 ± 0.02	
Stacking	Single layer	

Table S7. Comparison of electrochemical performance of reported high-voltage Graphite/LiCoO2 full cell configurations.

Modified strategies	Loading (mg cm^{-2})	Cell configuration	Rate (Current density (mA cm^{-2}))	Voltage range (V)	Cycle life	Residual capacity (mAh g^{-1})	Ref.
La, Al doping	10	coin cell	C/3 (0.90)	3.0-4.5	60	150.0	2
Hydrothermal assisted LAF-LCO	12.3-12.9	coin cell	0.1C (0.34)	3.0-4.5	70	155.6	3
Li_3PO_4 and $AlPO_4$ co-modified LCO	5	coin cell	0.3C (0.41)	3.0-4.5	100	174.1	4
Al+Ti bulk codoping + surface Mg doping	6.8-7.2	coin cell	0.5C (0.96)	3.0-4.5	200	162.4	5
Ti, Al, Mg co-doping	16.7-18.1	pouch cell	0.33C (1.57)	3.0-4.55	70	178.2	6
Coherent $LiMn_{1.5}Ni_{0.5}O_4$ coating	17	pouch cell	0.36C (1.70)	3.0-4.55	300	176.3	7
Surface Se treatment	16-17	pouch cell	0.36C (1.70)	2.95-4.57	450	/	8
Outside-in nanostructure fabracation	12.5-13.5	coin cell	0.3C (1.07)	3.0-4.55	86	146.6	This work
		pouch cell	0.2C (0.71)	3.0-4.5	135	156.1	

Supplementary References

[1] M. D. Radin, S. Hy, M. Sina, C. Fang, H. Liu, J. Vinckeviciute, M. Zhang, M. S. Whittingham, Y. S. Meng, A. Van der Ven, *Adv. Energy Mater*. **2017**, *7*, 1602888.

[2] Q. Liu, X. Su, D. Lei, Y. Qin, J. Wen, F. Guo, Y. A. Wu, Y. Rong, R. Kou, X. Xiao, F. Aguesse, J. Bareño, Y. Ren, W. Lu, Y. Li, *Nat. Energy* **2018**, *3*, 936.

[3] J. Qian, L. Liu, J. Yang, S. Li, X. Wang, H. L. Zhuang, Y. Lu, *Nat. Commun.* **2018**, *9*, 4918.

[4] X. Wang, Q. Wu, S. Li, Z. Tong, D. Wang, H. L. Zhuang, X. Wang, Y. Lu, *Energy Storage Mater*. **2021**, *37*, 67.

[5] L. Wang, J. Ma, C. Wang, X. Yu, R. Liu, F. Jiang, X. Sun, A. Du, X. Zhou, G. Cui, *Adv. sci*. **2019**, *6*, 1900355.

[6] J.-N. Zhang, Q. Li, C. Ouyang, X. Yu, M. Ge, X. Huang, E. Hu, C. Ma, S. Li, R. Xiao, W. Yang, Y. Chu, Y. Liu, H. Yu, X.-Q. Yang, X. Huang, L. Chen, H. Li, *Nat. Energy* **2019**, *4*, 594.

[7] Z. Zhu, D. Yu, Z. Shi, R. Gao, X. Xiao, I. Waluyo, M. Ge, Y. Dong, W. Xue, G. Xu, W.-K. Lee, A. Hunt, J. Li, *Energy Environ. Sci*. **2020**, *13*, 1865.

[8] Z. Zhu, H. Wang, Y. Li, R. Gao, X. Xiao, Q. Yu, C. Wang, I. Waluyo, J. Ding, A. Hunt, J. Li, *Adv. Mater.* **2020**, *32*, e2005182.
